# Why cultural distance can promote – or impede – group-beneficial outcomes

**DOI:** 10.1017/ehs.2024.8

**Published:** 2024-03-11

**Authors:** Bret Alexander Beheim, Adrian Viliami Bell

**Affiliations:** 1 Department of Human Behaviour, Ecology and Culture, Max Planck Institute for Evolutionary Anthropology, Leipzig, Germany; 2 Department of Anthropology, University of Utah, Salt Lake City, Utah, USA

**Keywords:** Cultural diversity, cultural similarity, *CF_ST_*, coordination, synergy

## Abstract

Quantifying the distance between cultural groups has received substantial recent interest. A key innovation, borrowed from population genetics, is the calculation of cultural *F_ST_* (*CF_ST_*) statistics on datasets of human culture. Measuring the variance between groups as a fraction of total variance, *F_ST_* is theoretically important in additive models of cooperation. Consistent with this, recent empirical work has confirmed that high values of pairwise *CF_ST_* (measuring cultural distance) strongly predict unwillingness to cooperate with strangers in coordination vignettes. As applications for *CF_ST_* increase, however, there is greater need to understand its meaning in naturalistic situations beyond additive cooperation. Focusing on games with both positive and negative frequency dependence and high-diversity, mixed equilibria, we derive a simple relationship between *F_ST_* and the evolution of group-beneficial traits across a broad spectrum of social interactions. Contrary to standard assumptions, this model shows why *F_ST_* can have both positive and *negative* marginal effects on the spread of group-beneficial traits under certain realistic conditions. These results provide broader theoretical direction for empirical applications of *CF_ST_* in the evolutionary study of culture.

## Graphical abstract

**Figure:**
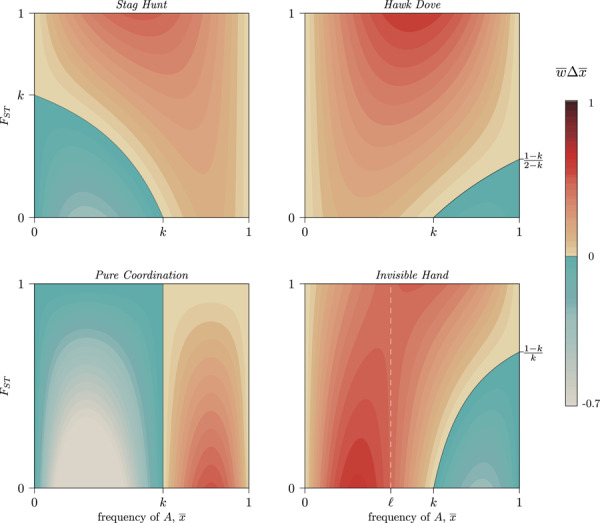

**Social media summary:** When should cultural distance between groups correlate – or not correlate – with parochial altruism and warfare?

## 1. Introduction

**Figure 1.**
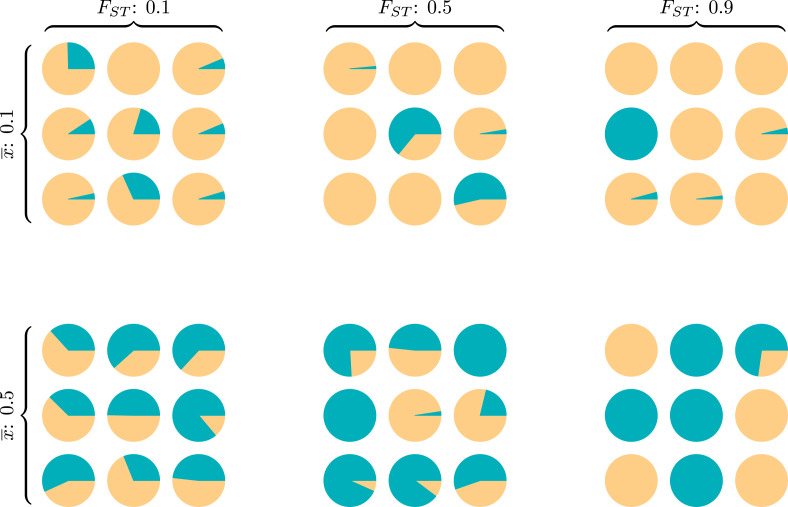
Six diversity scenarios for a metapopulation of nine groups (circles) characterised by a global mean 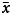 and *F_ST_*. For some discrete individual trait with two types, *A* (green) and *B* (yellow), we compare metapopulation mean frequencies of *A* at two levels, 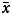 ∈{0.1,0.5} and three between-total variance ratios, *F_ST_* ∈{0.1,0.5,0.9}. Theoretical models of the evolution of cooperation indicate that metapopulations with equal *F_ST_* are equally likely to evolve altruistic behaviour, and altruism is most likely to evolve in metapopulations with higher *F_ST_* (rightmost column), regardless of 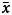 (Hamilton, 1975). The simulation code to reproduce this figure is in the Supporting Information.

Wright (1949) introduced *F_ST_* as a measure of genetic population structure to assess how genotype frequencies for each subpopulation differ from expectations assuming random mating. Also called the inbreeding coefficient, *F_ST_* responds to the relative influence of selection, migration, mutation and drift operating between and within groups (Holsinger & Weir, 2009). Like the well-known *R*^2^ or the *ICC* calculation in generalised linear modelling, the *F_ST_* index is a ratio of between-group to total variance, and measures the extent to which group structure ‘explains’ variation across a population on some discrete or continuous trait or set of traits. The popularity of such variance ratios is due in part to their ready interpretation – values near 0 indicate that traits within any specific group are about as variable as within the population as a whole, while values near 1 indicate that almost all variance exists between (mostly homogeneous) groups (Fig. 1).

Variance measures can similarly provide insights into cultural processes in group-structured populations. While the modes of cultural inheritance are more varied than in genetic evolution, cultural *F_ST_*, or *CF_ST_* (Bell et al., 2009), indicates the relative amount of segregation or self-assortment taking place on cultural traits, and can measure the between-group cultural distance caused by cultural selection, migration, social learning and other forces (Boyd & Richerson, 1985; Cavalli-Sforza & Feldman, 1981). The uses of cultural *F_ST_* have greatly multiplied in recent years, with study systems ranging from chimpanzee tool use (Boesch et al., 2020) to musical diversity (Rzeszutek et al., 2012), folk tales (Ross et al., 2013), the evolution of cooperation (Handley & Mathew, 2020; Smith et al., 2018) and cultural distances between religions (White et al., 2021) and nations (Muthukrishna et al., 2018). By answering what fraction of the total variance is found between groups, cultural measures of *F_ST_* mirror its broader use in genetics in understanding both the origins and implications of population structure.

In practice, *CF_ST_* is thought to positively predict the cooperativeness of groups. Smith et al. (2018) write that ‘if *F_ST_* is large enough, then individually deleterious but group-beneficial traits can evolve’ and Zefferman and Mathew (2015) argue that ‘a high cultural *F_ST_* promotes cultural predisposition for warfare because, as cultural norms and institutions are concentrated in specific groups, they will spread disproportionately as these groups win resources’.

In light of the recent popularity of *CF_ST_*, it is essential to strengthen the connection between *CF_ST_* and specific causal models of social evolution. To state that a *CF_ST_* estimate of 0.01, 0.1 or 0.6 is ‘large’ or ‘small’ is not provided by the Law of Total Variance, nor is the meaning of a comparison of *CF_ST_* values calculated on different traits or between two populations. Currently, the main justification for the use of *F_ST_* is its importance in mathematical models of cooperation, such as the Prisoner's Dilemma and the Public Goods Game, in which altruistic individuals pay some fixed cost to produce a fixed benefit within their group. In such additive models of the evolution of altruism, *F_ST_* becomes a concise measure of the relative scope for between-group and within-group selection (Hamilton, 1975), and serves as a quantitative threshold for the spread of altruistic behaviour, e.g. eq. (5) in Bowles (2006) and eq. (1) in both Bell et al. (2009) and Richerson et al. (2016) (see Supporting Information, SI, Section 1).

Yet, many features of social life are beyond the ability of such additive models of cooperation to articulate (Skyrms, 2004). In particular, the consequences for choosing a cooperative or non-cooperative behaviour are often not fixed, but rather depend on the current prevalence of behaviours within one's group; the interactions have *frequency-dependent* or *synergistic* payoffs (Grafen, 1979; Queller, 1985) and so lack dominant strategies. Many culturally transmitted norms have this property, and in such systems, the interests of the individual and the group are often not necessarily in opposition. For example, groups residing on opposite sides of an ecological or ethnic frontier often have institutions and cultural norms that are highly differentiated (McElreath et al., 2003), and so will have high pairwise *CF_ST_*. If social interactions in this meta-population resemble cooperative dilemmas, it is reasonable to expect the emergence of parochial altruism with culturally similar neighbours (Handley & Mathew, 2020) and even organised raiding and warfare against outgroups (Turchin, 2009; Zefferman & Mathew, 2015). Yet if social interactions were more accurately characterised by economic exchange, one might instead expect that potential access to non-local resources (Pisor & Gurven, 2016) or possible risk-buffering against local shocks (Liu & Mostafavi, 2023) would select for norms of generosity towards the out-group and greater cross-cultural competence (Bunce, 2020). Depending on the causal model of the social interaction, i.e. the ‘rules of the game’, the *CF_ST_* statistic may plausibly hold very different meanings. As with additive altruism, though, confirming such a hypothesis requires the careful reasoning provided by formal mathematical theory.

We here seek to develop the analytical connection between *F_ST_*, both cultural and genetic, and the evolution of group-beneficial traits (GBTs) across a variety of frequency-dependent social interactions. Our approach proceeds in three steps. First, we review the properties of various coordination and anti-coordination games to build a general frequency-dependent model of social interaction, from which games like Stag Hunt and Hawk–Dove can be viewed as specific cases. Our synthetic model of linear synergy builds on recent work by Allen and Nowak (2015) and Van Cleve (2017), and can describe a continuous spectrum of non-additive games via a single parameter, *θ*. Following the classic derivation of the evolution of altruism using the *F_ST_* variance ratio (Hamilton, 1975), we analyse our general frequency-dependent system to identify the conditions under which group-beneficial outcomes can evolve, focusing in particular on the role of within- and between-group variance. This extends classic results on *F_ST_* and assortment in mathematical biology (Gardner et al., 2011; Queller, 1985) into two regions of the synergistic spectrum not previously considered: simple coordination and complementarity. Based on these findings, we re-evaluate the existing body of empirical work on cultural *F_ST_* in light of some testable predictions from the model. By doing so we hope to pair the growing programme of quantifying cultural variation with a suite of models of social interaction framed in the language of evolutionary game theory.

## 2. The spectrum of social games

The field of evolutionary game theory has contributed substantially to our understanding of human and non-human societies over the last half-century (Gintis, 2000). In this approach, individual agents within a population are treated as expressing behavioural strategies which change in frequency through an evolutionary process, either the survival and reproduction of genetic alleles (Maynard Smith, 1982) or the social transmission of behaviours from demonstrators to learners (Smaldino, 2023). Fitness expressions that define the payoffs of specific strategies, both at the individual level and at the group level, are passed through an evolutionary replicator model. Behavioural options can themselves be treated as dichotomous choices (e.g. ‘cooperate’ or ‘defect’) or quantitative measures that fall on some continuum, e.g. allocations of resources between self and other. Interactions are commonly described as either pairwise, as a group of players forms dyads who each play the same two-player game together, or as a single *N*-person game in which all players contribute to shared payoffs. In all cases, a substantial amount of information about a social system can be encapsulated by asking whether a particular equilibrium state can be disrupted by intermittent or persistent shocks, and how different mechanisms of assortment and social structure affect the diffusion of cooperative traits, e.g. through reciprocity (Lehmann, Powers, & Schaik, 2022), punishment (Marlowe et al., 2008) or positive assortment via metapopulation segmentation (Taylor & Nowak, 2007). We here focus on games involving this latter mechanism, reviewing first the standard model of additive cooperation and then the different kinds of synergistic interactions which have been studied by game theorists.

### 2.1. Additive cooperation

In evolutionary biology, altruism refers to any behaviour that comes at a personal cost to the actor while benefiting others in a population. Much theoretical work on social evolution over the last half-century has focused on the conditions for the emergence of altruism, and has led to fruitful discoveries such as Hamilton's concept of inclusive fitness (Hamilton, 1964) and the gene's-eye perspective. Altruism is also important to the study of population genetics, because it is perhaps the simplest possible representation of a conflict between group and individual interests, and so provides an extreme test of the properties of specific population structures (Rogers, 1990). In both of these contexts, *F_ST_* plays a central role.

In an additive model of altruism in group-structured populations, we imagine that the *i*th individual in the *j*th group has a phenotype *x_ij_* between 0 and 1, representing their propensity towards cooperation. If *x_ij_* = 1, then this individual pays a fitness cost *c* to create a collective benefit *b* for all members of their group (including themselves). Individuals in the group who are not altruistic (*x_ij_* = 0) pay no such cost, but experience the group benefits from other altruists. Formally, we can represent the fitness payoff for an individual as
1
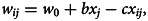

where *c* and *b* are the cost and benefits of altruism, and *x_j_* is the mean frequency of altruism within group *j*. Note that Eq. (1) does not require that interactions be dyadic, and applies to both pairwise Prisoner's Dilemma-type interactions and *N*-player Public Goods Games (SI Section 1).

Extending the original concept of relatedness by shared ancestry in his famous rule, Hamilton (1975) found that the strength of selection for altruistic behaviours in such a system is directly proportional to the fraction of behavioural variance that exists between groups, such that altruism can evolve under the condition
2
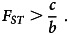

This result is a general feature of linear, additive interactions in structured populations, because the *F_ST_* statistic serves as a complete summary of the extent to which altruists positively assort with one another (Taylor & Nowak, 2007), as genetic relatedness does in systems of interacting kin. This selection threshold is empirically useful, because it suggests that observed *F_ST_* values in additive interactions will be strongly correlated with the prevalence of cooperative behaviour. Consistent with this, Handley and Mathew (2020) have shown that Kenyan pastoralists are more willing to cooperate with hypothetical strangers who are more culturally similar to them, as measured by *CF_ST_* between the respective ethnic groups, and Smith et al. (2018) find high *CF_ST_* across Hadza camps in contributions to multi-person Public Goods Game experiments. As many mechanisms of human cultural transmission can maintain relatively high between-group variation (Boyd & Richerson, 1985), and observed *F_ST_* ratios for human groups are generally much larger for cultural than genetic traits (Bell et al., 2009), the high degree of parochial altruism observed in human societies is plausibly understood via the transmission of cooperative norms structured by human cultural groups (Richerson et al., 2016; Zefferman & Mathew, 2015).

### 2.2. Four categories of non-additive games

The use of *CF_ST_* as a quantitative measure of cultural diversity is motivated by its prominence in evolutionary models of altruism, so its significance depends on the extent that real-world social interactions resemble the underlying assumptions of Eq. (1). Yet this model has been criticised for being unrepresentative of most social interactions (Alvard & Nolin, 2002; Skyrms, 2004). While theories of altruism posit a fundamental opposition between the interests of a group and of individuals within the group, many real-world institutions function to *align* individual and group incentives by adjusting reward structures, e.g. through punishment (Marlowe et al., 2008; Molleman et al., 2019) or reciprocity (Lehmann et al., 2022; Panchanathan & Boyd, 2004).

Further, models of cooperation such as the Prisoner's Dilemma usually assume an additive payoff structure, such that the marginal cost to an individual of switching their behaviour to altruism is the same whether one is in a group entirely of altruists or in a group entirely of defectors, or any mixture of the two. In many naturalistic contexts, though, a social behaviour's consequences are a function of how common it is within the population. Cutting a queue may bring angry, immediate sanctioning in a group where it is rare, but, where common, be a self-reinforcing way to organise turn-taking, or even a social norm. Conversely, an unusual strategy in a competition, or novel product in a marketplace, may derive its success primarily by its rarity vs. commonplace alternatives. As a result, the best response in each situation is always dependent on the behaviours of others, and so there is no dominant strategy. Many kinds of social interaction are non-additive, and linguistic variation, music, sartorial traits and other domains of culture are better described by other game-theoretic models, e.g. Stag Hunt, Snowdrift, Chicken or Hawk–Dove (Camerer, 2003; Gintis, 2000; Maynard Smith, 1982; Skyrms, 2004; Smaldino, 2023). These games can all be characterised by the presence of frequency-dependent or synergistic payoffs, and can be divided broadly into four categories.

#### 2.2.1. Simple coordination

Individuals benefit from coordinating on the same behaviour in many kinds of social interaction. The success of a rowing team in a race, a troupe of dancers, the flow of a traffic system or the functioning of a code of laws often depends on participants all doing exactly the same thing, possibly in the presence of a coordinating authority. In coordination games, the worst outcomes are generally experienced by mixed groups in which different participants act with incompatible behaviours, i.e. groups with high behavioural diversity. The origins of norm psychology (House et al., 2020), economic agglomeration (Krugman, 1991), by-product mutualisms (Hauert et al., 2006) and positive network externalities (Katz & Shapiro, 1985; Liebowitz & Margolis, 1994; Schelling, 1973) are fundamentally rooted in coordination.

In such interactions, a marginal increase in a norm, behaviour or strategy (generically, a ‘trait’) within a group will increase the payoffs of individuals using that trait, and reduce the payoffs of those not using that trait. To distinguish this class of interactions from coordination dilemmas (described below), we refer to these as *simple coordination* games, although they have also been called ‘correlative coordination’ (Smaldino, 2023), ‘relaxed’ social dilemmas (Allen & Nowak, 2015) or just ‘coordination’ (Cooney, 2022).

In many cases of simple coordination, the choice between alternatives is functionally arbitrary, such as the decision to drive on the left or right side of the road, or to adopt purely symbolic markers of group identity (McElreath et al., 2003). These can be described by the Pure Coordination game, represented by the 2 × 2 payoff matrix:

**Table:** 

	*A*	*B*
*A*	5	3
*B*	2	5

Here, the payoffs are given for the ‘row-player’, who receives this amount given their trait and the trait of their partner, the ‘column-player’ (whose payoffs are symmetrical). Although the usual framing involves two players making a simultaneous choice following this payoff matrix, Pure Coordination-type interactions can just as easily apply to large groups or entire societies (SI Section 2.3). Regardless of the framing scenario, the essential detail in Pure Coordination is that all players receive the same high payoff if they can successfully coordinate on one of the two equivalent alternatives.

In other kinds of coordination, the two options instead have a clear difference in performance which has consequences for between-group competition. A common example is differing norms about acceptable marriage partners, which may impact the size and cohesion of political alliances (Schulz et al., 2018). Following Boyd and Richerson (2002), we refer to these better-performing traits as group-beneficial traits (GBTs), because the group receives a higher payoff at one pure-strategy equilibrium vs. the other. Group-beneficial traits exists in most synergistic interactions (with exceptions such as Pure Coordination), but the GBT pure-strategy equilibrium is not always the group-optimum trait distribution. The existence of GBT's is important in a structured metapopulation, as groups that coordinate on different equilibria can compete with one another through equilibrium selection (Bowles, 2006; Richerson et al., 2016).

#### 2.2.2. Coordination dilemmas

In contrast to simple coordination, a coordination *dilemma* exists when all individuals experience a coordination dynamic but, regardless of their own behaviour, benefit from the increased prevalence of the GBT. This could be because one of the two options produces some kind of public good which all individuals in a group benefit from (Boyd & Richerson, 2002), or, alternatively, the other trait produces some kind of ‘public bad’ which is costly to all individuals. Within game theory, the most famous coordination dilemma is the Stag Hunt, initially described by Jean-Jacques Rousseau (Skyrms, 2004), usually defined by a 2 × 2 payoff matrix such as

**Table:** 

	Stag	Hare
Stag	5	0
Hare	2	2

As Rousseau put it, hunters are better off coordinating to hunt a stag to earn the highest payoff, but may be tempted to hunt hare instead, abandoning their stag-hunting partners who earn nothing as a result. In this classic formulation, a Hare player receives the same mediocre payoff regardless of their partner's behaviour, so technically the Stag Hunt represents a boundary between simple coordination and a coordination dilemma. Like cooperative dilemmas, much research has focused on circumstances under which group-beneficial traits spread within coordination dilemmas. Although individuals are incentivised to choose the GBT, risk-averse players may require assurance that their partner will also, and for this reason coordination dilemmas are also called ‘assurance games’ (Sen, 1967). Coordination dilemmas as a group are also often referred to generically as ‘stag hunts’ (Cooney, 2022; Taylor & Nowak, 2007; Van Cleve, 2017), although different games have different characteristics with respect to equilibrium selection (Boyd & Richerson, 2002). Here, we reserve the term ‘Stag Hunt’ to refer exclusively to the algebraic form of the specific game structure above (SI Section 3), rather than coordination dilemmas as a category.

#### 2.2.3. Anti-coordination dilemmas

Another important class of games involves a negative frequency dependence between strategies. As in coordination dilemmas, an anti-coordination dilemma requires that all individuals benefit from the increased prevalence of the GBT. However, unlike coordination dilemmas, each trait can realise higher payoffs within a group composed of the other trait, so individuals are always incentivised to play the rare strategy. Since neither strategy is stable against invasions by the other, evolutionary systems involving anti-coordination dilemmas tend to approach a stable mixture of strategies, but this mixed equilibrium always realises lower benefits than those of the group-optimum configuration of strategies.

Anti-coordination dilemmas are often described in terms of negative externalities or congestion games (Peña & Nöldeke, 2023), and a popular anti-coordination dilemma is Snowdrift (Doebeli & Hauert, 2005). Here, two individuals are trying to accomplish a group project that produces a shared benefit (e.g. a village well, a road clear of snow or a co-authored manuscript) but each has the temptation to shirk their part in the labour. This game is described algebraically by benefit *b* and cost 0 *< c < b*, such that the row-player's payoff table is

**Table:** 

	Work	Shirk
Work	*b* − *c*/2	*b* − *c*
Shirk	*b*	0

As is generally the case in anti-coordination dilemmas, payoff-maximising individuals would prefer to live in a group in which all other players are Workers, but can realise a higher payoff by individually defecting to ‘Shirk’. However, this negative frequency dependence works in both directions: a group of Shirkers pays a very large cost (producing no group project), so the cooperative Work strategy is also able to invade.

Another important example of an anti-coordination dilemma is Maynard Smith's (1982) Hawk–Dove game, defined by payoff matrix

**Table:** 

	Dove	Hawk
Dove	*V*/2	0
Hawk	*V*	(*V* − *C*)/2

for resource *V* and cost of fighting *C > V*. If both agents employ the Dove strategy, each has an equal chance of getting the resource. If one plays Hawk and the other Dove, the Hawk gets all the resources without a fight, and the Dove nothing. If both play Hawk, though, a fight begins in which one gains the resource at a large cost to the other, again with equal chance to each participant. Overt conflict is the worst outcome, both for groups and for individuals, and as a result each strategy can invade the other when it is rare.

Although derived independently in very different contexts, Hawk–Dove and Snowdrift have essentially identical evolutionary dynamics. Anti-coordination dilemmas as a whole are often referred to in this literature as ‘hawk–doves’ (Cooney, 2022; Taylor & Nowak, 2007; Van Cleve, 2017) or ‘snowdrifts’ (Allen & Nowak, 2015). As above, though, we reserve the terms Snowdrift and Hawk–Dove to refer specifically to the original payoff matrices defined above, distinguishing them from other games in the larger, heterogeneous class of anti-coordination interactions.

#### 2.2.4. Complementarity

Not all forms of anti-coordination are antagonistic. In Lamaleran whale hunting, success depends on the coordinated actions of not only a harpooner, a bailer and a helmsman, but also a sailmaker, a carpenter and a blacksmith (Alvard & Nolin, 2002). Likewise, the specialised roles within an ant colony, an orchestra, a sports team or a sailing crew, or the production and flow of goods and services within a marketplace, depend on behavioural diversity. Because each behaviour experiences a higher payoff when rare, this is essentially an anti-coordination dynamic, but unlike anti-coordination dilemmas above, neither trait benefits from a marginal increase in itself within the group; an increase in the abundance of each trait is always beneficial to individuals choosing the alternative. For this reason, we refer to this category of interaction as *complementarity*.

Following Adam Smith's famous metaphor, we define the Invisible Hand game as the complementary interaction in which the group-optimal frequency of behaviours is also the mixed equilibrium itself (see SI Section 3.3). An example payoff matrix for this game might be

**Table:** 

	*A*	*B*
*A*	5	6
*B*	6	2

As a kind of mirror-image of simple coordination, players here can realise the highest payoffs by individually specialising in one or the other trait to generate a synergistic payoff. In economics, complementarity is most famously associated with the principles of ‘gains from trade’ and Ricardian comparative advantage, and in sociology and evolutionary biology, with divisions of labour (Cooper et al., 2021). Confusingly, the phrase ‘strategic complements’ is also used in game theory to describe coordination games, in the sense that two players both using the same trait may generate positive synergies when interacting. Here, we use the concept of complementarity strictly to refer to *different* behaviours, traits or strategies ‘complementing’ each other.

Some kinds of complementarity may give the same outcome to all participants regardless of who does what, and as with coordination, which behaviour is chosen by which participant may be totally arbitrary. Depending on their role in the interaction, however, each participant may earn different payoffs, which allows complementarity to serve as a model for studying the origins of inequality and unfairness (O'Connor, 2019).

## 3. A model of linear synergy with mixed equilibria

Although the categories of interaction described above have different characteristics, and the games within those categories are derived from different scenarios, they are all connected by synergistic or frequency-dependent payoffs and (with notable exceptions like the Pure Coordination game) by the presence of GBTs. Unlike additive cooperation, there is no dominant strategy in any such games, as each player's best option always depends on what their partners do. As a result, it is valuable to consider all these interactions simultaneously in the context of a general model. To abstract away from specific framing scenarios such as ‘cooperation’, ‘defection’, ‘hunt stag’ and ‘hunt hare’, etc., we instead define two generic behavioural strategies, *A* and *B*, and interpret each phenotype *x_ij_* as a propensity toward trait *A*, taking any real value between 0 and 1, inclusive (which strategy to track is arbitrary). As before, we define a structured population with group-average phenotype *x_j_* for each group *j* and individual phenotype *x_ij_* for each individual *i* in group *j*.

To derive a tractable model that incorporates the above phenomena, we make two assumptions. As in the metapopulation model in Eq. (1), we assume that payoffs to all individuals are linear with respect to the group frequency of *A*, *x_j_*. Define parameter *m* as the marginal effect of a within-group increase in trait *A* on the fitness of a focal individual with *A*, and *n* as the marginal effect of such an increase for a focal individual with trait *B*. For individuals with mixed strategies the marginal effect of an increase in *A* is *x_ij_m* + (1 − *x_ij_*)*n*, the weighted average of *m* and *n*. We assume that *m* and *n* are both constant over possible distributions of behaviour within a group, but make no assumption about their signs or relative magnitudes. The assumption of linearity is critical, as non-linear fitness functions require higher-order moments to evaluate the covariance between individual and group phenotype, and *F_ST_* will no longer be sufficient to describe group structure (Schonmann & Boyd, 2016). One limitation of our approach, however, is that by treating group fitness as a simple average of over individual (linear) fitness, it ignores the concept of viscosity or local competition (Hamilton, 1964).

Second, in order to incorporate the concept of frequency dependence, we assume non-additivity or synergy with respect to individual frequency of behaviour, such that a marginal change in *x_ij_* has a different impact on individual payoff as a function of group frequency *x_j_*. This is distinct from the concept of linearity, by which we mean that a change in *x_j_* has a constant marginal effect on those in the group, regardless of the group frequency *x_j_*. Thus, a model can be both linear and non-additive. Synergistic effects imply that an individual increasing their use of behaviour *A* can sometimes decrease, and sometimes increase their personal fitness depending on how common *A* is in the group, so there must be some group frequency *k* at which this individual effect is zero. Together, these assumptions produce the fitness expression
3
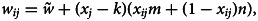

for real numbers *m*, *n* and 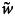 (see Table 2 for a complete list of symbols). This fitness expression describes pairwise interactions whose payoffs depend on the group frequency of traits, but also *N*-player interactions in which users of each strategy experience constant per capita returns to scale (Peña et al. (2015), detailed in SI Section 2.3).

Like additive altruism in Eq. (1), groups of individuals playing synergistic games experience two pure-strategy equilibria, but each of the games described above are distinguished by the presence of a third, mixed equilibrium *k* at which different strategies co-exist with the same payoffs within a group. Depending on the interaction structure, this mixed-strategy equilibrium may be stable or unstable. In coordination games, *k* separates the two basins of attraction for each pure-strategy equilibrium. Harsanyi and Selten (1988) define the risk-dominant equilibrium as having the larger basin, which is more likely to be reached by stochastic evolutionary dynamics (Kandori et al., 1993; Young, 1993). In anti-coordination games, in contrast, *k* represents the stable equilibrium whose basin of attraction covers *x_j_* ∈ (0, 1), and in complementarity games, groups at *k* have higher payoffs than at either of the two pure-strategy equilibria (SI Section 3.3).

In general, we do not require *k* to be between 0 and 1, and synergistic games can exist without a third, mixed equilibrium. We can also express Eq. (3) as
4
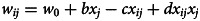

which extends Eq. (1) with a synergistic coefficient *d* ∈ **R**. In the above notation, *m* = *b* + *d* while *n* = *b*, and all members of a group experience the same fitness payoff at group frequency *k* = *c*/*d*. Assuming further that *b >* 0, *c >* 0, and *c* − *b < d < c*, this formulation describes the ‘Prisoner's Dilemma with synergy’ (Ohtsuki, 2012; Van Cleve, 2017).

We do not use this parameterisation in this analysis, because we want to be as vague as possible about the causal mechanics of traits *A* and *B* in order to more easily interpret different synergistic dynamics that might resemble Eq. (3). Our model covers games in which *A* is GBT, in the sense that a group of *A* will have a higher average fitness than a group of *B* (−*nk < m*(1 − *k*)), but also games in which *B* is GBT (−*nk > m*(1 − *k*)) and interactions like Pure Coordination, in which neither trait is GBT (−*nk* = *m*(1 − *k*)). The specific details of different interactions may indeed resemble a Prisoner's Dilemma, such as the choice between conserving or over-harvesting a local marine resource, which benefits one individual at the expense of others. However, there are many synergistic interactions in which the concepts of ‘cooperation’ and ‘defection’ do not make sense, such as the choice between using LaTeX or Microsoft Word to write co-authored articles. Here, payoffs are influenced by the ambient number of users within one's collaboration networks, and each alternative works well when commonplace.

### 3.1. Mapping the four categories of interaction

The general model of linear synergy described by Eq. (3) can define the four categories of synergistic interaction simply by whether the marginal effects *m* and *n* are, respectively, either positive or negative. Simple coordination interactions require that an increase in trait *A* benefits those with *A* (*m >* 0) and harms those with trait *B* (*n <* 0), while in complementarity the reverse is true (*m <* 0, *n >* 0). A coordination dilemma occurs when an increase in the GBT has a positive effect on all, but the marginal benefit is larger for those with the GBT (that is, *m > n >* 0 if *A* is GBT). Anti-coordination dilemmas, in contrast, require that the benefit of increasing the GBT is larger for those without the GBT, and *vice versa*, as each trait has a higher payoff when rare.

We are hardly the first to map this space of interactions, or to recognise that different games can be related to each other by transformation through a continuous spectrum. Modelling groups within a cultural metapopulation, Boyd and Richerson (2002) use a similar approach to differentiate coordination dilemmas by the strength of selection and size of the relative basins of attraction. Hauert et al. (2006) and Taylor and Nowak (2007) show how the Prisoner's Dilemma can be transformed algebraically into other games, defining anti-coordination and coordination dilemmas using inequalities between absolute payoffs. This approach is further developed by Van Cleve (2017) to incorporate the concepts of synergy and reciprocity, while Allen and Nowak (2015) and Cooney (2022) extend this notation to cover complementarity and simple coordination interactions.

**Figure 2.**
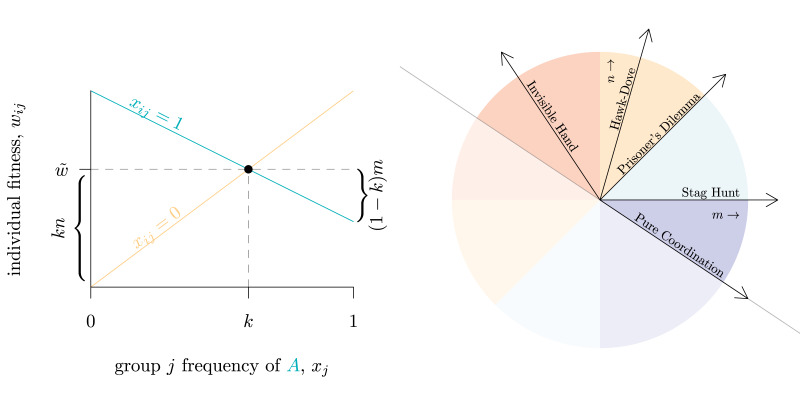
(left) Payoffs for frequency-dependent interactions, for individuals who only employ trait *A* (*x_ij_* = 1, green) and only employ trait *B* (*x_ij_* = 0, yellow) following Eq. (3). In this particular interaction, *n* = −*mk*/(1 − *k*), which defines the Invisible Hand game with *k* = 0.6. (right) Phase space of all possible games described by Eq. (3), with well-known game structures defined by specific ratios (slopes) of *n* to *m*. Here *k* = 0.6. Coordination dilemmas in which *A* is GBT (light blue) are defined by *m* > *n* > 0, and simple coordination by *m* > 0, *n* < 0 (dark blue). Complementarity games exist whenever *m <* 0, *n >* 0 (red) and anti-coordination dilemmas in which *A* is GBT by *n > m >* 0 (yellow). The space is symmetrical about the line *n* = −*m*(1 − *k*)/*k* so only the top half of the space is annotated (the bottom half is much the same, except *B* is now the GBT). The non-synergistic Prisoner's Dilemma exists at the degenerate case in which *m* = *n* (see SI Section 2)

We see our approach here as complementary with existing methods. For the broad group of games with mixed equilibria (0 *< k <* 1), one advantage of our parameterisation is the ability to articulate the differences between games with the same *k* with only two terms, *m* and *n*. The (*m*, *n*) space then forms a kind of map for all possible interactions in this model (Fig. 2, right). This is because the specific value of parameter *k* is not important for the properties of the interactions relevant to our analysis (provided it remains between 0 and 1); a Hawk–Dove game with *k* = 0.1, *k* = 0.5, or *k* = 0.9 is still a Hawk–Dove game per its definition above (Maynard Smith, 1982). By interpreting trait *A* as ‘Dove’ and trait *B* as ‘Hawk’, the Hawk–Dove dynamic is captured in this model when *m* = *V*/2 and *n* = (*V* + *C*)/2. At Dove frequency *k* = (*C* − *V*)/*C*, all individuals have the same average fitness, so it holds that

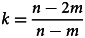

meaning that, in (*m*, *n*) space, Hawk–Dove-like interactions exist for any game in which

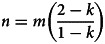

where *n >* 0 and *m >* 0 and where 0 *< k <* 1.

**Figure 3.**
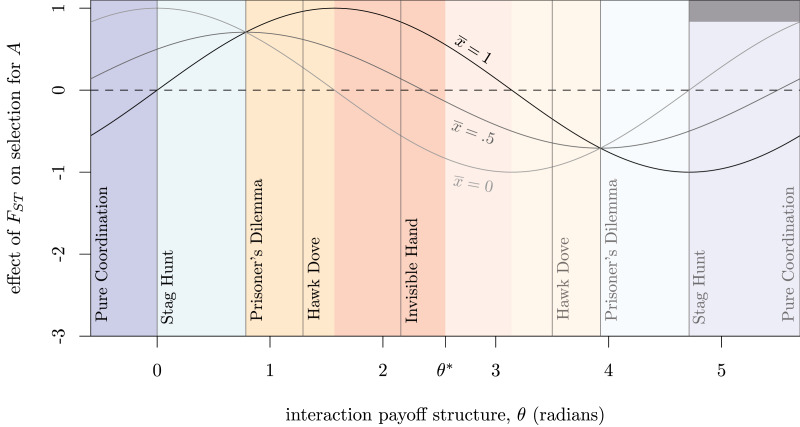
Marginal effects of an increase in *F_ST_* on the spread of *A* across the spectrum of linear game structures following Eq. (8) with *k* = 0.6 and three values of 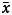. Effect units are given by d*w*Δ*x*/d*F_ST_* × var(*x_j_*)^−1^. Named games are located at specific points on the spectrum, with colours corresponding to the four regions described in Fig. 2, right. Trait *A* is a GBT over the left half of the spectrum (until θ* = atan2((1 − *k*), −*k*)), and *B* is a GBT in the lighter right half. No GBT exists at Pure Coordination and θ*.

We can supply a similar analysis for any coordination or anti-coordination game with a defined payoff structure, and derive a characteristic relationship between *m*, *n*, and *k* in the general model above (Table 1, examples). As a result, for a given *k*, specific games will appear as vectors within the (*m*, *n*) space (Fig. 2, right), and all vectors with the same slope are effectively the ‘same’ game. This means that to differentiate games with the same equilibrium frequency *k*, we only need one parameter, the polar angle *θ*, where tan*θ*= *n*/*m*. This gives us the ability to articular *all* linear synergistic interactions with the same mixed equilibrium along a single numerical scale. Doing so identifies that several canonical games exist at critical locations within this space marking transitions between regions (Fig. 3).

**Table 1.** Categories of linear synergy in terms of the marginal effect *m* of an increase in the frequency of *A* on individuals with trait *A* and marginal effect *n* on individuals with trait *B*, with specific examples of each category defined in terms of *m*, *n* and equilibrium frequency *k*. The conditions given for both coordination dilemmas and anti-coordination dilemmas assume that trait *A* is a group-beneficial trait (GBT), and equivalent conditions exist if instead *B* is a GBT. Pure Coordination, in contrast, requires that neither trait is a GBT. Note the example games assume also the mixed equilibrium is attainable, i.e. 0 < *k* < 1. See SI Section 3 for complete derivations of each game condition.

Interaction category	Model conditions
Simple coordination	*m >* 0, *n <* 0
e.g. Pure Coordination	*n* = −*m*(1 − *k*)/*k*
Coordination dilemmas	*m > n* ≥ 0
e.g. Stag Hunt	*m >* 0, *n* = 0
Anti-coordination dilemmas	*n > m* ≥ 0
e.g. Hawk–Dove	*n* = *m*(2 − *k*)/(1 − *k*)
Complementarity	*m <* 0, *n >* 0
e.g. Invisible Hand	*n* = −*mk*/(1 − *k*)

## 4. The role of ***F_ST_*** in the spread of group-beneficial traits

Having defined the model of linear synergy, we now seek an expression similar to that of Hamilton (1975) that illuminates the role of *F_ST_* in the spread of group-beneficial traits. In a metapopulation model, group interactions structure payoffs to individuals through frequency-dependent feedback, so one reasonable method to analyse such a model is by the multi-level version of the Price equation, which partitions covariance dynamics within and between groups as
5
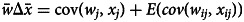

Although initially developed to describe genetic evolution, the Price equation can be equally applied to the decomposition of distinct processes of cultural transmission (Beheim & Baldini, 2012; El Mouden et al., 2014), including group-level traits (Smaldino, 2014) and gene–culture coevolutionary systems (Aguilar & Akçay, 2018). Supplying our model of frequency dependence and assuming dichotomous phenotypes, we show in the Appendix that this expression becomes
6
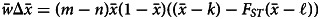

where payoff ratio ℓ = *m*/(*m* − *n*). The critical variance ratio at which the group-beneficial trait neither spreads or declines (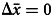) is then
7
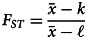

This threshold separates *F_ST_* values that cause an increase in group-beneficial traits from those that cause a decrease, but the specific causal details of the system (defined by payoff ratio ℓ and equilibrium location *k*) determine both the size and direction of evolutionary change. We now also require the metapopulation prevalence of *A*, 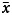, which was not present in the additive condition of Eq. (2).

We can assess the effect of a marginal increase in *F_ST_* on selection for *A* by taking the derivative of Eq. (6) with respect to *F_ST_*,
8
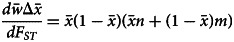

When *A* is the GBT (–*nk < m*(1 – *k*)), positive values of this selection gradient indicate stronger selection for the GBT as *F_ST_* increases, while negative values imply greater selection against the GBT. If trait *B* is instead the GBT (-*nk > m*(1 − *k*)), the reverse is true. Because 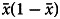 is necessarily positive, this phenomenon is thus mediated entirely by the signs and relative magnitudes of *m* and *n*, with the selection gradient reversing direction at 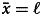. We can see the behaviour of Eq. (8) graphically over the four categories of interaction in Fig. 3 by graphing the arctan *θ* of *m* and *n*.

**Figure 4.**
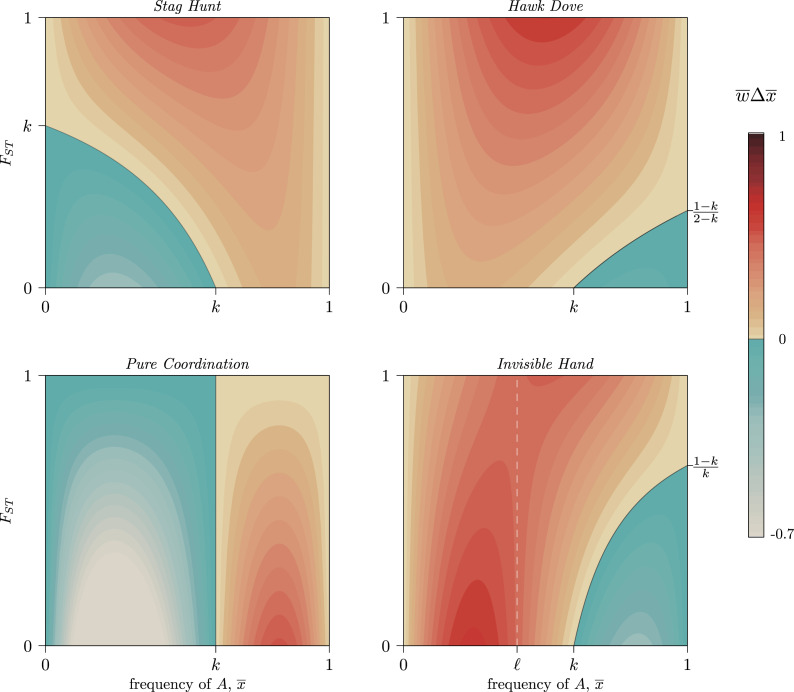
Contour levels (colouration) showing the strength of selection on a generic trait *A* in four frequency-dependent games with *k* = 0.6 per Eq. (6). Trait *A* can increase at any frequency, 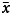, provided that metapopulation *F_ST_* exceeds the critical value set by Eq. (7). Stag Hunt and Hawk–Dove both show increasing selection for the GBT as *F_ST_* increases (a positive marginal effect). Pure Coordination, on the other hand, shows a uniformly negative marginal effect, and Invisible Hand has a negative marginal effect of *F_ST_* below, and positive marginal effect above, the frequency 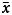 = ℓ (white dashed line) per Eq. (8). See SI Section 3 for detailed descriptions of each game. A simple coordination game with a similar pattern to Invisible Hand is described in SI Section 3.4, following Allen and Nowak (2015).

In coordination or anti-coordination dilemmas, *F_ST_* must have a positive marginal effect on selection for group-beneficial trait, for the same reason Hamilton (1975) described: at high levels of *F_ST_*, behaviours that raise average group payoffs can positively assort with one another, thereby avoiding some costs from free riding. For example, in a Stag Hunt, a high *F_ST_* enables groups at different pure-strategy equilibria to compete with one another through direct conflict (Bowles, 2006), differential dispersion (Rogers, 1990) or social influence (Boyd & Richerson, 2002) (Fig. 4, *Stag Hunt*). Consistent with this, Kenyan pastoralists who frequently engage in intergroup conflict show a strong association between pairwise *F_ST_* and willingness to engage with partners in coordination vignettes (Handley & Mathew, 2020). In anti-coordination dilemmas such as Hawk–Dove, high *F_ST_* indicates the ability for group-beneficial traits like Dove to avoid interacting with group-harmful behaviours like Hawk. If *F_ST_* is high enough, this positive assortment on like-type can prevent Hawk from invading a population altogether (Fig. 4, *Hawk–Dove*). This positive selection gradient is also present in coordination dilemmas and anti-coordination dilemmas without a third, mixed equilibrium (*k <* 0 or *k >* 1) such as the ‘Prisoner's Dilemma with synergy’ (SI Section 2.1).

Outside of coordination and anti-coordination dilemmas, however, we can see in Fig. 3 how *F_ST_* can both facilitate or hinder the spread of a group-beneficial trait depending on 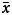. Because the reversal of the selection gradient with respect to *F_ST_* lies at frequency ℓ, it can only occur in complementarity and simple coordination interactions if 0 < *k* < 1, because only they allow 0 *<* ℓ *<* 1. In these regions, 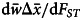 will be positive for some 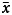 and negative for others. When the two coordination alternatives are essentially arbitrary, *F_ST_* slows movement through each basin of attraction towards a pure-strategy equilibrium, as some groups will coordinate on the minority norm even as it declines in the population overall (Fig. 4, *Pure Coordination*). Similarly, complementary strategies spread faster when *F_ST_* is low, as they can more quickly find unlike-types, while with high *F_ST_* each trait can become stuck inside low-diversity behavioural enclaves that cannot realise the full benefits of complementarity (Fig. 4, *Invisible Hand*).

Although directly relevant to the study of cultural *F_ST_*, this phenomenon has not been clearly identified in either the cultural or social evolution literature to date. The role of *F_ST_* in models of linear synergy was initially described for discrete traits by Queller (1985), and our Eq. (6) is isomorphic with Eq. (10) in Gardner et al. (2011) and Eq. (A33) in Lehmann et al. (2008). We can also re-express Eq. (7) in terms of Queller's (1985) ‘synergy coefficient’ *d* as

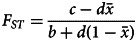

In this version, it is clear that if there are no synergistic effects (*d* = 0), this simplifies back to Hamilton's (1975) well-known threshold (Van Cleve & Lehmann, 2013). Because our approach reduces the essential differences between games with the same *k* down to a single parameter, *θ*, we can more readily connect the role of *F_ST_* to each of the four regions of interaction via Eq. (8) and Fig. 3. Allen and Nowak (2015) report that positive assortment between genetic relatives (which is analogous to *F_ST_*) can inhibit coordination when *m* + *n <* 0, assuming *A* is GBT, and provide an example game at *k* = 1/7, *θ* = −1.19 (also see SI Fig. A13). Taking a different approach with Eq. (8), we find that this inhibitory phenomenon is much more general, and appears whenever 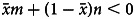 (assuming *A* is GBT) or 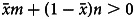 (assuming *B* is GBT). For games with mixed equilibria (0 < *k* < 1), cultural distance can slow the spread of GBTs under certain trait frequencies throughout both the simple coordination (*m >* 0, *n <* 0) and complementarity (*m <* 0 and *n >* 0) regions of the interaction spectrum.

## 5. Discussion

Our results challenge the prevailing view in cultural evolution that cultural distance between groups, as measured by *CF_ST_*, should positively associate with the prevalence of parochial altruism, homophily preferences, between-group warfare and so forth. Although this finding has been empirically validated, our model results suggest that it should be viewed as valid only within a specific range of a broader spectrum of interaction. Within certain game structures – complementarity and simple coordination – we can rather predict the opposite, that group-beneficial traits spread slower, or are prevented from spreading altogether, when cultural distance between groups is relatively high and within-group trait diversity is low (i.e. *CF_ST_* is high). These results suggest that properly designed experimental or observational studies should be able to show *heterophily*, a preference for interacting with those different from one's in-group.

Thus, because the causal details of frequency-dependent interactions (as defined by *m*, *n* and *k*) determine the effect of *F_ST_* on the evolution of group-beneficial traits, caution when interpreting and comparing empirical *CF_ST_*s is warranted. Even within a game such as Stag Hunt, in which higher values of *F_ST_* promote the spread of group-beneficial Stag behaviours, the presence of synergistic effects complicates the ability to compare distance measures across different systems. This is fundamentally because the population average plays a mediating role in Eq. (7). As a result, paradoxically, a large *CF_ST_* might indicate *weaker* selection for group-beneficial traits while a small *CF_ST_* indicates *stronger* selection (Fig. 4, *Stag Hunt*). Meta-analyses cataloging observed cultural *F_ST_* values would therefore benefit from contextualising these estimates with both the mean prevalence of the behaviours they are tracking and from careful descriptions of the causal details of each system under comparison.

By making explicit the relationship between *F_ST_* and the outcomes of frequency-dependent interactions, we not only better-situate empirical measures of cultural distance, but also motivate new theoretical inquiry into questions around synergy, frequency-dependence, network measures, the complexities of identity, the paradox of diversity and other questions that heavily rely on the structure of variation across groups. As such, these results can bridge a number of disparate literatures on social evolution, cultural evolution, graph theory and group identity.

### 5.1. Dichotomous and continuous traits

**Figure 5.**
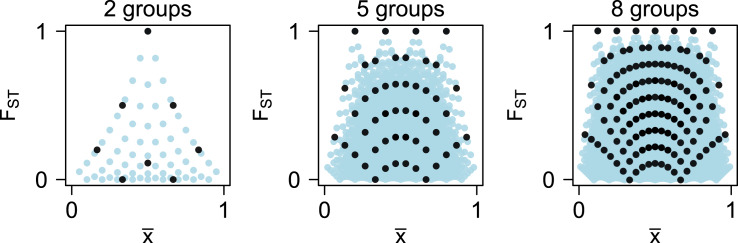
For a metapopulation of *M* groups each of *N* individuals with binary traits, there are 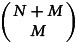 unique combinations of 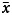 and *F_ST_*. Shown are possible values for groups of *N* = 3 individuals (black) and *N* = 10 individuals (blue).

Our derivation (Appendix A) generalises Queller's (1985) original analysis of dichotomous traits over finite numbers of groups and individuals, allowing us to consider the effects of selection over the full range of possible values of *F_ST_* via Eq. (A3). Assuming discrete traits allows us to simplify the system to Eq. (6), but this imposes combinatoric constraints on the possible values of 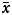 and *F_ST_*. With *M* groups each with *N* individuals, for example, there are 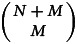 possible combinations of 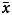 and *F_ST_*, with systematic under-representation of corner cases as a function of both *M* and *N* (Fig. 5). This indicates that if *F_ST_* values are calculated pairwise between two groups (*M* = 2), high *F_ST_* values cannot be reached at low or high values of 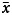 regardless of group size. With even moderate numbers of groups and individuals, though, most of the possible range of both 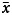 and *F_ST_* is reachable. Thus, Eq. (6) and the resulting selection surfaces (Fig. 4) are best understood in the context of a large number of large groups.

### 5.2. Networks, identities and the complexities of population structure

Our result was derived in the context of a purely hierarchical population, in which each individual has unambiguously one, and only one, group membership. Yet in realistic social settings, individuals often have multiple overlapping group identities. While we expect that the qualitative features of anti-coordination, coordination and complementarity described here are quite general, we also believe that decomposing the multiple memberships may illuminate the relevant variance measures in empirical applications.

Specifically, we may decompose the first covariance in Eq. (5) relating trait value and average group fitness, by asking how an individual's trait value covaries with the multiple groups to which they may belong. If the identities or groups are not competing or mutually exclusive, their dynamics may be treated independently, i.e. a separate Eq. (5) for each group. However, if groups affect each other in some way, we will require a system of *N* equations for *N* identities. In such a case the relevant variance measures will reflect the properties of a system of equations, such as stationary distributions if an equilibrium exists, or a cyclical dynamic otherwise. This may be an important theoretical avenue to pursue.

We may also consider a network approach, where we can decompose the first term in Eq. (5) into *conditional* covariances. That is, the covariance of a trait value and its fitness is conditional on another random variable, which in the network context may be the strength of the tie between two individuals with a certain phenotype. The key statistic – the expectation of conditional covariances across network ties – in general reflects assortment mechanisms central to the literature around group-beneficial traits, and the relevant empirical variance measures will be expressed through parameters prescribing assortment according to individual trait value.

### 5.3. Resolving the paradox of cultural diversity

Our results also provide theoretical focus to a persistent empirical debate about the relationship between immigration, multiculturalism, assimilation and trust, recently framed as the ‘paradox of diversity’ (Schimmelpfennig et al., 2021). A substantial literature in sociology and political science has shown that racial and ethnic diversity at the neighbourhood level is associated with decreased levels of generalised trust (Dinesen & Sønderskov, 2015) and consequently a decline in civic engagement (Alesina et al., 2001; Putnam, 2007). Yet, at the same time, the integration of marginalised minorities into a market system is often associated with an *increase* in expression of pro-social preferences (Henrich et al., 2010) and willingness to trust advice from co-ethnic strangers (Lightner & Hagen, 2021), and in urbanising economies local immigration rates have been positively associated with increases in wages (Ottaviano & Peri, 2006) and rates of innovation (Posch et al., 2023).

Since *F_ST_* serves as an indicator of cultural homogeneity within groups, our model serves as a simple demonstration of this paradox. Consistent with sociological findings, low *CF_ST_* (i.e. high within-group diversity) erodes the positive assortment necessary to sustain a group-beneficial trait in anti-coordination dilemmas, e.g. Hawk–Dove, or to bootstrap it in coordination dilemmas, e.g. Stag Hunt. However, when interactions are complementary, a low *CF_ST_* more rapidly promotes socially-optimal outcomes. This mirrors the empirical pattern of higher marginal gains from specialisation in diverse urban economies (Peri & Sparber, 2009; Posch et al., 2023). Moreover, in simple coordination interactions, higher within-group diversity moves a population faster towards a social optimum, as it becomes harder for some subpopulations to become ‘stuck’ within the basin of attraction of an inferior normative equilibrium. Cultural diversity can have opposite effects in different causal interaction structures. As with the parable of the blind men describing different parts of an elephant, we should expect such contradictory findings to make more sense when placed in the larger context of the spectrum of synergistic interactions (Fig. 3).

### 5.4. From statics to dynamics

To date much of the theoretical literature has focused on evaluating broad questions, such as when cooperation will evolve. Therefore, analytical attention has been drawn to assessing the evolutionary scope of a trait via static analysis, e.g. Bell et al. (2009), which is also the approach used here. As a result, we do not track the change in *F_ST_* as we do the change in mean frequency 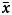. This is unsatisfactory when wishing to predict trait variation and selection over time, and in future models, special attention should be made to the relationship between traits and their inheritance, the multilevel dynamics of selection over time, and how to put them together to estimate the parameters of a particular case (Keller, 1999).

For cultural traits, understanding the individual-level transmission of a particular trait is key as many inheritance pathways are possible which may affect the covariance between trait values and the ‘next-generation’ learner. Likewise, the transmission of group-level traits may occur through multiple mechanisms, including selective imitation, migration and natural selection (Richerson et al., 2016). Given the diverse transmission mechanisms and other evolutionary forces, it is likely that selection on groups, institutions, individuals or other units may evolve at different time scales. As a result, *CF_ST_* will change through time and consequently so will the predicted rate and perhaps direction of selection. A dynamic approach also requires more detailed consideration of stochasticity and long-run stability (Foster & Young, 1990). In coordination interactions, Van Cleve and Lehmann (2013) show that if selection is relatively weak, the relative size of each basin of attraction, random mutation and positive assortment together determine which trait reaches long-run fixation.

## 6. Conclusion

We formalise the causal significance of *F_ST_* across synergistic social interactions through a game theoretic frame, better justifying its use in diverse cultural contexts. Our results demonstrate that there is no single relationship between *CF_ST_* and the strength of selection for group-beneficial traits. With the increasing popularity of *CF_ST_* and similar measures of behavioural diversity between groups, our analysis strongly motivates increased attention to the ethnographic contexts affecting cultural variation. Doing so will give greater power to empirical variance measures for inferring or reflecting underlying causal mechanisms.

## Supporting information

Beheim and Bell supplementary material Beheim and Bell supplementary material

## Data Availability

n/a

## Supplementary material

To view supplementary material for this article, please visit https://doi.org/10.1017/ehs.2024.8

## Research transparency and reproducibility

No data were directly used in this paper. R code to reproduce all figures and calculations is available at https://github.com/babeheim/causality-variance-fst.

## Appendix: Evolutionary Decomposition

In a metapopulation in which individual *i* in group *j* has phenotype *x_ij_* and experiences fitness payoff *w_ij_*, behaviour *A* will increase according to

where *w_j_* and *x_j_* are the mean fitness and average phenotypic frequency within group *j*. Note that these are *empirical* covariances and expectations calculated over a finite number of groups. A full glossary of mathematical symbols is found in Table 2. Define φ = *n*/(*n* − *m*), so that from Eq. (3) individual fitness becomes

Within any particular group, the covariance between individual fitness and phenotype can be written as

which, because cov(φ, *x*_*ij*_) = 0, becomes

Substituting this into the second term of Eq. (5) gives

A1

On the group level, the mean fitness can be written as

so the covariance between group fitness and group phenotype simplifies to

A2

Combining Eq. (A1) and Eq. (A2), the full expression for evolutionary change in mean phenotype is

Define ℓ = 1 − φ and write  as the regression coefficient of on *x*_*j*_, then evolutionary change in our frequency-dependent model is given as

A3

Equation (A3) holds for continuous phenotypes (*x*_*ij*_ ∈ [0, 1]) as a frequency-dependent extension of Hamilton's Eq. (2). Previous work generally assumes that traits are discrete 0/1 variables, e.g. Eq. (10) in Gardner et al. (2011), Eq. (A33) in Lehmann et al. (2008) and Appendix D in Allen and Nowak (2015). In this special case in which individual phenotypes have dichotomous traits (*x*_*ij*_ ∈ {0, 1}), then ,  and *β* = 1. Equation (A3) thus simplifies to

which is Eq. (6) in the main text. If −*nk* < *m*(1 − *k*), then positive values of this expression indicate that the GBT (trait *A*) will be selected for, and negative values indicate that the GBT will be selected against. If −*nk* > *m*(1 − *k*), then *B* is the GBT and the reverse is true.

**Table 2.** Glossary of variables used

Variable	Description
*x*_*ij*_	Quantitative phenotype of individual *i* in group *j*
*w*_*ij*_	Fitness of individual *i* in group *j*
*x*_*j*_	Average phenotype in group *j*
*w*_*j*_	Average fitness in group *j*
	Average phenotype in the population
	Average fitness in the population
*F*_*ST*_	= var(*x_j_*)/var(*x*), between-group to total phenotypic variance
*CF*_*ST*_	*F_ST_* calculated on cultural traits, variants, or beliefs
*b*	Group benefit from cooperative individual phenotype
*c*	Individual cost from cooperative individual phenotype
*d*	Synergistic effect from cooperative individual phenotype
*m*	Marginal effect of a group increase in *A*, *x_j_*, on an individual with trait *A*
*n*	Marginal effect of a group increase in *A*, *x_j_*, on an individual with trait *B*
ℓ	= *m*/(*m* − *n*), group frequency of trait *A* where the selection gradient reverses
φ	= *n*/(*n* − *m*) = 1 − ℓ
*θ*	= atan2(*n*, *m*), the polar angle of a line segment with slope *n*/*m*
*k*	Group frequency of trait *A* at which all individual fitnesses are equal
	Fitness for all individuals in group *j* at *x_j_* = *k*

## References

[ref1] Aguilar, E. G., & Akçay, E. (2018). Gene–culture coinheritance of a behavioral trait. The American Naturalist, 192(3), 311–320. doi: 10.1086/698872 30125232

[ref2] Alesina, A., Glaeser, E., & Sacerdote, B. (2001). *Why doesn't the US have a European-style welfare system?* National Bureau of Economic Research. doi: 10.3386/w8524

[ref3] Allen, B., & Nowak, M. A. (2015). Games among relatives revisited. Journal of Theoretical Biology, 378, 103–116. doi: 10.1016/j.jtbi.2015.04.031 25953388

[ref4] Alvard, M., & Nolin, D. (2002). Rousseau's whale hunt? Coordination among big-game hunters. Current Anthropology, 43(4), 533–559. doi: 10.1086/341653

[ref5] Beheim, B. A., & Baldini, R. (2012). Evolutionary decomposition and the mechanisms of cultural change. Cliodynamics, 3, 18.

[ref6] Bell, A. V., Richerson, P. J., & McElreath, R. (2009). Culture rather than genes provides greater scope for the evolution of large-scale human prosociality. Proceedings of the National Academy of Sciences, 106(42), 17671–17674. doi: 10.1073/pnas.0903232106 PMC2764900 19822753

[ref7] Boesch, C., Kalan, A. K., Mundry, R., Arandjelovic, M., Pika, S., Dieguez, P., …, Kuhl, H. S. (2020). Chimpanzee ethnography reveals unexpected cultural diversity. Nature Human Behaviour, 4(9), 910–916. doi: 10.1038/s41562-020-0890-1 32451479

[ref8] Bowles, S. (2006). Group competition, reproductive leveling, and the evolution of human altruism. Science, 314(5805), 1569–1572. doi: 10.1126/science.1134829 17158320

[ref9] Boyd, R., & Richerson, P. J. (1985). Culture and the evolutionary process. Chicago, IL: University of Chicago Press.

[ref10] Boyd, R., & Richerson, P. J. (2002). Group beneficial norms can spread rapidly in a structured population. Journal of Theoretical Biology, 215(3), 287–296. doi: 10.1006/jtbi.2001.2515 12054837

[ref11] Bunce, J. A. (2020). Field evidence for two paths to cross-cultural competence: Implications for cultural dynamics. Evolutionary Human Sciences, 2, e3. doi: 10.1017/ehs.2020.1 37588369 PMC10427313

[ref12] Camerer, C. (2003). Behavioral game theory: Experiments in strategic interaction. Princeton, NJ: Princeton University Press.

[ref13] Cavalli-Sforza, L. L., & Feldman, M. W. (1981). Cultural transmission and evolution: A quantitative approach. Princeton University Press. 7300842

[ref14] Cooney, D. B. (2022). Assortment and reciprocity mechanisms for promotion of cooperation in a model of multilevel selection. Bulletin of Mathematical Biology, 84(11), 126. doi: 10.1007/s11538-022-01082-8 36136162

[ref15] Cooper, G. A., Frost, H., Liu, M., & West, S. A. (2021). The evolution of division of labour in structured and unstructured groups. eLife, 10, e71968. doi: 10.7554/eLife.71968 34713804 PMC8789276

[ref16] Dinesen, P. T., & Sønderskov, K. M. (2015). Ethnic diversity and social trust: Evidence from the micro-context. American Sociological Review, 80(3), 550–573. doi: 10.1177/0003122415577989

[ref17] Doebeli, M., & Hauert, C. (2005). Models of cooperation based on the Prisoner's Dilemma and the Snowdrift game: Prisoner's dilemma and the snowdrift game. Ecology Letters, 8(7), 748–766. doi: 10.1111/j.1461-0248.2005.00773.x

[ref18] El Mouden, C., Andre, J.-B., Morin, O., & Nettle, D. (2014). Cultural transmission and the evolution of human behaviour: A general approach based on the Price equation. Journal of Evolutionary Biology, 27(2), 231–241. doi: 10.1111/jeb.12296 24329934

[ref19] Foster, D., & Young, P. (1990). Stochastic evolutionary game dynamics. Theoretical Population Biology, 38(2), 219–232. doi: 10.1016/0040-5809(90)90011-J

[ref20] Gardner, A., West, S. A., & Wild, G. (2011). The genetical theory of kin selection. Journal of Evolutionary Biology, 24(5), 1020–1043. doi: 10.1111/j.1420-9101.2011.02236.x 21371156

[ref21] Gintis, H. (2000). Game theory evolving. Princeton, NJ: Princeton University Press.

[ref22] Grafen, A. (1979). The hawk–dove game played between relatives. Animal Behaviour, 27, 905–907. doi: 10.1016/0003-3472(79)90028-9

[ref23] Hamilton, W. D. (1964). The genetical evolution of social behavior, I & II. Journal of Theoretical Biology, 7, 1–52. 5875341 10.1016/0022-5193(64)90038-4

[ref24] Hamilton, W. D. (1975). Innate social aptitudes of man: An approach from evolutionary genetics. In R. Fox (Ed.), Biosocial anthropology (pp. 133–155). London: Malaby Press.

[ref25] Handley, C., & Mathew, S. (2020). Human large-scale cooperation as a product of competition between cultural groups. Nature Communications, 11(1), 702. doi: 10.1038/s41467-020-14416-8 PMC7000669 32019930

[ref26] Harsanyi, J. C., & Selten, R. (1988). A general theory of equilibrium selection in games. Cambridge, MA: MIT Press.

[ref27] Hauert, C., Michor, F., Nowak, M. A., & Doebeli, M. (2006). Synergy and discounting of cooperation in social dilemmas. Journal of Theoretical Biology, 239(2), 195–202. doi: 10.1016/j.jtbi.2005.08.040 16242728 PMC2891160

[ref28] Henrich, J., Ensminger, J., McElreath, R., Barr, A., Barrett, C., Bolyanatz, A., …, Ziker, J. (2010). Markets, religion, community size, and the evolution of fairness and punishment. Science, 327(5972), 1480–1484. doi: 10.1126/science.1182238 20299588

[ref29] Holsinger, K. E., & Weir, B. S. (2009). Genetics in geographically structured populations: Defining, estimating and interpreting FST. Nature Reviews Genetics, 10(9), 639–650. doi: 10.1038/nrg2611 PMC4687486 19687804

[ref30] House, B. R., Kanngiesser, P., Barrett, H. C., Broesch, T., Cebioglu, S., Crittenden, A. N., …, Silk, J. B. (2020). Universal norm psychology leads to societal diversity in prosocial behaviour and development. Nature Human Behaviour, 4(1), 36–44. doi: 10.1038/s41562-019-0734-z 31548679

[ref31] Kandori, M., Mailath, G. J., & Rob, R. (1993). Learning, mutation, and long run equilibria in games. Econometrica, 61(1), 29–56. doi: 10.2307/2951777

[ref32] Katz, M. L., & Shapiro, C. (1985). Network Externalities, Competition, and Compatibility. The American Economic Review, 75(3), 424–440.

[ref33] Keller, L. (1999). Levels of selection in evolution. Princeton, NJ: Princeton University Press.

[ref34] Krugman, P. (1991). Increasing returns and economic geography. The Journal of Political Economy, 99(3), 483–499.

[ref35] Lehmann, L., Feldman, M., & Foster, K. (2008). Cultural transmission can inhibit the evolution of altruistic helping. The American Naturalist, 172(1), 12–24. doi: 10.1086/587851 18500938

[ref36] Lehmann, L., Powers, S. T., & Schaik, C. P. v. (2022). Four levers of reciprocity across human societies: Concepts, analysis and predictions. Evolutionary Human Sciences, 4. doi: 10.1017/ehs.2022.7 PMC10426116 37588908

[ref37] Liebowitz, S. J., & Margolis, S. E. (1994). Network externality: An uncommon tragedy. Journal of Economic Perspectives, 8(2), 133–150. doi: 10.1257/jep.8.2.133

[ref38] Lightner, A. D., & Hagen, E. H. (2021). Acculturation and market integration are associated with greater trust among Tanzanian Maasai pastoralists. Evolutionary Human Sciences, 3. doi: 10.1017/ehs.2021.10 PMC10427282 37588557

[ref39] Liu, C.-F., & Mostafavi, A. (2023). Revealing hazard-exposure heterophily as a latent characteristic of community resilience in social-spatial networks. Scientific Reports, 13(1), 4817. doi: 10.1038/s41598-023-31702-9 36964245 PMC10039027

[ref40] Marlowe, F. W., Berbesque, J. C., Barr, A., Barrett, C., Bolyanatz, A., Cardenas, J. C., …, Tracer, D. (2008). More ‘altruistic’ punishment in larger societies. Proceedings of the Royal Society B: Biological Sciences, 275(1634), 587–592. doi: 10.1098/rspb.2007.1517 PMC2596817 18089534

[ref41] Maynard Smith, J. (1982). Evolution and the theory of games. Cambridge: Cambridge University Press. doi: 10.1017/CBO9780511806292

[ref42] McElreath, R., Boyd, R., & Richerson, P. (2003). Shared norms and the evolution of ethnic markers. Current Anthropology, 44(1), 122–130. doi: 10.1086/345689

[ref43] Molleman, L., Kolle, F., Starmer, C., & Gächter, S. (2019). People prefer coordinated punishment in cooperative interactions. Nature Human Behaviour, 3(11), 1145–1153. doi: 10.1038/s41562-019-0707-2 31477909

[ref44] Muthukrishna, M., Bell, A., Henrich, J., Curtin, C., Gedranovich, A., McInerney, J., & Thue, B. (2018). Beyond WEIRD psychology: Measuring and mapping scales of cultural and psychological distance. SSRN Electronic Journal. doi: 10.2139/ssrn.3259613 PMC7357184 32437234

[ref45] O'Connor, C. (2019). The origins of unfairness: Social categories and cultural evolution. Oxford: Oxford University Press.

[ref46] Ohtsuki, H. (2012). Does synergy rescue the evolution of cooperation? An analysis for homogeneous populations with non-overlapping generations. Journal of Theoretical Biology, 307, 20–28. doi: 10.1016/j.jtbi.2012.04.030 22579553

[ref47] Ottaviano, G. I., & Peri, G. (2006). The economic value of cultural diversity: Evidence from US cities. Journal of Economic Geography, 6(1), 9–44. doi: 10.1093/jeg/lbi002

[ref48] Panchanathan, K., & Boyd, R. (2004). Indirect reciprocity can stabilize cooperation without the second-order free rider problem. Nature, 432(7016). doi: 10.1038/nature02978 15565153

[ref49] Peña, J., & Nöldeke, G. (2023). Cooperative dilemmas with binary actions and multiple players. OSF Preprints. doi: 10.31219/osf.io/8y2z5

[ref50] Peña, J., Nöldeke, G., & Lehmann, L. (2015). Evolutionary dynamics of collective action in spatially structured populations. Journal of Theoretical Biology, 382, 122–136. doi: 10.1016/j.jtbi.2015.06.039 26151588

[ref51] Peri, G., & Sparber, C. (2009). Task specialization, immigration, and wages. American Economic Journal: Applied Economics, 1(3), 135–169. doi: 10.1257/app.1.3.135

[ref52] Pisor, A. C., & Gurven, M. (2016). Risk buffering and resource access shape valuation of out-group strangers. Scientific Reports, 6(1), 30435. doi: 10.1038/srep30435 27470126 PMC4965756

[ref53] Posch, M., Schulz, J., & Henrich, J. (2023). Surname diversity, social ties and innovation. SSRN Scholarly Paper, Rochester, NY. doi: 10.2139/ssrn.4531209

[ref54] Putnam, R. D. (2007). Diversity and community in the twenty-first century. Scandinavian Political Studies, 30(2), 38.

[ref55] Queller, D. C. (1985). Kinship, reciprocity and synergism in the evolution of social behaviour. Nature, 318(6044), 366–367. doi: 10.1038/318366a0

[ref56] Richerson, P., Baldini, R., Bell, A. V., Demps, K., Frost, K., Hillis, V., …, Zefferman, M. (2016). Cultural group selection plays an essential role in explaining human cooperation: A sketch of the evidence. Behavioral and Brain Sciences, 39, e30. doi: 10.1017/S0140525X1400106X 25347943

[ref57] Rogers, A. R. (1990). Group selection by selective emigration: The effects of migration and kin structure. American Naturalist, 135, 398–413.

[ref58] Ross, R. M., Greenhill, S. J., & Atkinson, Q. D. (2013). Population structure and cultural geography of a folktale in Europe. Proceedings of the Royal Society B: Biological Sciences, 280(1756), 20123065. doi: 10.1098/rspb.2012.3065 PMC3574383 23390109

[ref59] Rzeszutek, T., Savage, P. E., & Brown, S. (2012). The structure of cross-cultural musical diversity. Proceedings of the Royal Society B: Biological Sciences, 279(1733), 1606–1612. doi: 10.1098/rspb.2011.1750 PMC3282333 22072606

[ref60] Schelling, T. C. (1973). Hockey helmets, concealed weapons, and daylight saving: A study of binary choices with externalities. Journal of Conflict Resolution, 17(3), 381–428. doi: 10.1177/002200277301700302

[ref61] Schimmelpfennig, R., Razek, L., Schnell, E., & Muthukrishna, M. (2021). Paradox of diversity in the collective brain. Philosophical Transactions of the Royal Society B: Biological Sciences, 377(1843), 20200316. doi: 10.1098/rstb.2020.0316 PMC8666911 34894736

[ref62] Schonmann, R. H., & Boyd, R. (2016). A simple rule for the evolution of contingent cooperation in large groups. Philosophical Transactions of the Royal Society B: Biological Sciences, 371(1687), 20150099. doi: 10.1098/rstb.2015.0099 PMC4760199 26729938

[ref63] Schulz, J., Bahrami-Rad, D., Beauchamp, J., & Henrich, J. (2018). The origins of WEIRD psychology. SSRN Scholarly Paper, Rochester, NY. doi: 10.2139/ssrn.3201031

[ref64] Sen, A. K. (1967). Isolation, assurance and the social rate of discount. The Quarterly Journal of Economics, 81(1), 112–124. doi: 10.2307/1879675

[ref65] Skyrms, B. (2004). The stag hunt and the evolution of social structure. Cambridge: Cambridge University Press.

[ref66] Smaldino, P. E. (2014). The cultural evolution of emergent group-level traits. Behavioral and Brain Sciences, 37(3), 243–254. doi: 10.1017/S0140525X13001544 24970399

[ref67] Smaldino, P. E. (2023). Modeling social behavior: Mathematical and agent-based models of social dynamics and cultural evolution. Princeton, NJ: Princeton University Press.

[ref68] Smith, K. M., Larroucau, T., Mabulla, I. A., & Apicella, C. L. (2018). Hunter–gatherers maintain assortativity in cooperation despite high levels of residential change and mixing. Current Biology, 28(19), 3152–3157.e4. doi: 10.1016/j.cub.2018.07.064 30245106

[ref69] Taylor, C., & Nowak, M. A. (2007). Transforming the dilemma. Evolution, 61(10), 2281–2292.doi: 10.1111/j.1558-5646.2007.00196.x 17711471 PMC2396516

[ref70] Turchin, P. (2009). A theory for formation of large empires. Journal of Global History, 4(2), 191–217. doi: 10.1017/S174002280900312X

[ref71] Van Cleve, J. (2017). Stags, hawks, and doves: Social evolution theory and individual variation in cooperation. Integrative and Comparative Biology, 57(3), 566–579. doi:10.1093/icb/icx071 28957516

[ref72] Van Cleve, J., & Lehmann, L. (2013). Stochastic stability and the evolution of coordination in spatially structured populations. Theoretical Population Biology, 89, 75–87. doi:10.1016/j.tpb.2013.08.006 23999503

[ref73] White, C. J. M., Muthukrishna, M., & Norenzayan, A. (2021). Cultural similarity among coreligionists within and between countries. Proceedings of the National Academy of Sciences, 118(37). doi: 10.1073/pnas.2109650118 PMC8449328 34493675

[ref74] Wright, S. (1949). The genetical structure of populations. Annals of Eugenics, 15(1), 323–354. doi: 10.1111/j.1469-1809.1949.tb02451.x 24540312

[ref75] Young, H. P. (1993). The evolution of conventions. Econometrica, 61(1), 57–84. doi:10.2307/2951778

[ref76] Zefferman, M. R., & Mathew, S. (2015). An evolutionary theory of large-scale human warfare: Group-structured cultural selection. Evolutionary Anthropology: Issues, News, and Reviews, 24(2), 50–61. doi: 10.1002/evan.21439 25914359

