# Supplementary material for: Why cultural distance can promote – or impede – group-beneficial outcomes
Source: Evol Hum Sci. 2024 Mar 11;6:e14. doi: 10.1017/ehs.2024.8 (PMC10955364; doi:10.1017/ehs.2024.8)
Supplement: Beheim and Bell supplementary material [file S2513843X24000082sup001.pdf]

Supplementary Materials for "Why cultural distance can promote — or impede — group-beneficial outcomes"

Bret Alexander Beheim*[1] and Adrian Viliami Bell[2]

[1]Department of Human Behavior, Ecology and Culture, Max Planck Institute for Evolutionary Anthropology, Leipzig, Germany
[2]Department of Anthropology, University of Utah, Salt Lake City, USA

1 The evolution of additive altruism

1.1 The role of F_{ST} in linear, additive interactions

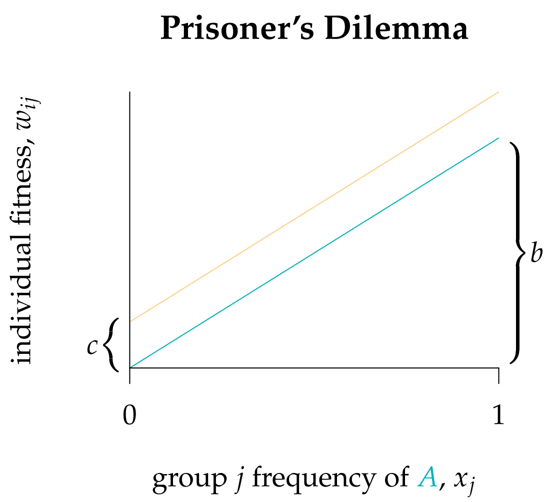

Figure A1: Payoff functions for the Prisoner's Dilemma.

*bret_beheim@eva.mpg.de

In a linear cooperative interaction, the expected payoff for each individual i in group j is

$$w_{ij} = w_0 - cx_{ij} + bx_j \tag{A1}$$

where x_{ij} and x_j are the individual and group phenotype, respectively. Parameter b represents the benefit all individuals in the group experience from the presence of cooperative phenotypes, while c represents the individual cost for their cooperative behavior, where $b > c > 0$.

Because we assume individuals exist within distinct groups, we can employ the multilevel Price equation to partition total evolutionary change into the covariance dynamics within and between groups as

$$\overline{w}\Delta\overline{x} = \mathrm{cov}(w_j, x_j) + \mathrm{E}(\mathrm{cov}(w_{ij}, x_{ij})). \tag{A2}$$

With linear effects of phenotype on fitness, this expression can be simplified to

$$\overline{w}\Delta\overline{x} = (b - c)\mathrm{var}(x_j) - c\mathrm{E}(\mathrm{var}(x_{ij}))$$

where $\mathrm{var}(x_j)$ represents the between-group variance in mean phenotype and $\mathrm{E}(\mathrm{var}(x_{ij}))$ represents the average variance within each group. Since the total variance in the metapopulation, $\mathrm{var}(x)$, is equal to the sum of these two quantities per the Law of Total Variance,

$$= b\,\mathrm{var}(x_j) - c\left(\mathrm{var}(x_j) + \mathrm{E}(\mathrm{var}(x_{ij}))\right)$$

$$= b\,\mathrm{var}(x_j) - c\,\mathrm{var}(x)$$

Re-expressed with $F_{ST} = \mathrm{var}(x_j)/\mathrm{var}(x)$, the difference equation becomes

$$\overline{w}\Delta\overline{x} = \mathrm{var}(x)(b\,F_{ST} - c) \tag{A3}$$

Thus, altruism will increase in frequency whenever $b\,F_{ST} > c$, or when $F_{ST} > c/b$ (Fig. A2). Since the F_{ST} variance ratio is bounded between 0 and 1, the assumption that $b > c$ is crucial (Hamilton, 1975).

Taking the derivative of Equation (A3) with respect to F_{ST} yields

$$\frac{\mathrm{d}\overline{w}\Delta\overline{x}}{\mathrm{d}F_{ST}} = \mathrm{var}(x)\,b$$

This quantity is necessarily positive, so a marginal increase in within-group diversity (a.k.a. a decrease in F_{ST}) will slow the rate at which cooperative behaviors can spread at any frequency $\overline{x}$.

1.2 Linear, additive fitness in pairwise and N-person games

What kind of interactions give rise to the fitness expression of Eq. (A1)? The default scenario in evolutionary game theory is the random pairing of agents in a simultaneous,

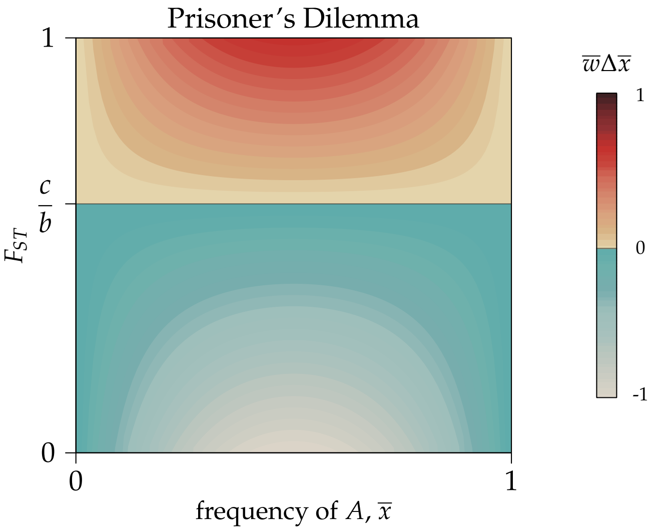

Figure A2: Contour levels (coloration) showing the strength of selection on spread of the cooperative trait A in a metapopulation playing Prisoner's-Dilemma-like cooperative dilemmas per main text Eq. (1). Altruism will spread at any frequency, $\overline{x}$, provided the metapopulation $F_{ST} > c/b$.

symmetric game with the classic payoffs of the additive Prisoner's Dilemma (Table A1). If focal individual i is paired with alter j, their payoff is then given by

$$w_i = w_0 + x_i(x_j(b-c) + (1-x_j)(-c)) + (1-x_i)x_j b$$

$$= w_0 + x_j b - x_i c$$

If the way that such dyads form is unspecified, x_j can be treated as a random variable from individual i's perspective, and the unconditional expectation of x_j is by assumption the group frequency of A ($E(x_j) = p$). Thus, the *expected* fitness payoff for individual i is Eq. (A1).

	A	B
A	$b-c$	$-c$
B	b	0

Table A1: Row-player payoffs in a Prisoner's Dilemma, where $b > c > 0$.

Linear fitness expressions are not confined to just dyadic interactions. In the linear Public Goods Game, we imagine that each individual i contributes benefit $x_i b$ into a group production function, at a personal cost of cx_i. The resulting aggregate benefits are bX, where $X = \sum_{i=1}^{N} x_i$, and because this is a public good, each individual receives bX/N regardless of their individual contribution. Thus, the individual fitness function is

$$w_i = w_0 + b\frac{X}{N} - cx_i.$$

Since p is the average frequency of behavior A in the population,

$$p = \frac{1}{N}\sum_{i=1}^{N} x_i = \frac{X}{N},$$

we have again reached Eq. (A1), but without explicitly assuming dyadic or pairwise payoffs in the manner of a Prisoner's Dilemma, nor that it represents an expectation given an unknown partner. The linear PGG is commonly used to represent a non-excludable, ambient benefit from an individual's cooperative activities, e.g. picking up trash in a public park, or forgoing exploitation of a fishery or forest (Ostrom, 2003). Coefficient b is known commonly as the "multiplier" in a PGG, representing the marginal aggregate return-on-investment for a given amount of activity (Gavrilets & Richerson, 2017). In this model, the marginal effect of an individual's contribution to their fitness, $\mathrm{d}w_i/\mathrm{d}x_i$, does not depend on the contributions of others, so there is no possibility of frequency-dependence or synergistic effects.

2 The evolution of frequency-dependent group-beneficial traits

2.1 Adding synergy to the Prisoner's Dilemma

A simple way to capture the concept of synergistic or frequency-dependent effects is a modification of the additive Prisoner's Dilemma model above. Assume that two cooperators interacting together produce a total benefit which is either greater than, or less than, the sum of their individual contributions, represented by some real value d. The revised payoff matrix is given in Table A2.

	A	B
A	$b - c + d$	$-c$
B	b	0

Table A2: Row-player payoffs in a Prisoner's Dilemma with synergy, where $b > c > 0$ and $c - b < d < c$.

For a population whose average tendency to play A is p, if any individual i plays strategy A x_i of the time, their expected payoff w_i would be

$$w_i = w_0 + x_i(p(b - c + d) + (1 - p)(-c)) + (1 - x_i)pb \tag{A4}$$

where w_0 gives a baseline payoff for all players. Re-arranging yields

$$w_i = w_0 + bp - cx_i + dpx_i \tag{A5}$$

which simplifies to the additive PD expression when $d = 0$. The synergy coefficient thus serves as an interaction term between the focal's tendency towards A and the expected tendency of their partner. Supplying this into the Price equation yields an evolution difference equation for trait A of

$$\overline{w}\Delta\overline{x} = \overline{x}(1 - \overline{x})(bF_{ST} - c + d(\overline{x} + F_{ST}(1 - \overline{x})) \tag{A6}$$

Taking the derivative with respect to F_{ST} yields

$$\frac{\mathrm{d}\overline{w}\Delta\overline{x}}{\mathrm{d}F_{ST}} = \overline{x}(1 - \overline{x})(b + d(1 - \overline{x})) \tag{A7}$$

As before, the marginal effect of F_{ST} on selection will always be positive within this range, and group segmentation will allow A to evolve so long as

$$F_{ST} > \frac{c - d\overline{x}}{b + d(1 - \overline{x})}$$

Given that $b > c > 0$, this game qualitatively resembles an additive Prisoner's dilemma so long as $c - b < d < c$. If the synergistic benefit is very large ($d > c$), a mixed equilibrium emerges at group frequency $k = c/d$, and the game becomes a coordination dilemma. If, on

the other hand, the synergistic effect is very deleterious ($d < c - b$), then a group of A has a lower fitness than a group of B, so A is not a GBT and the interaction is no longer a social dilemma at all (Taylor & Nowak, 2007).

One way to extend this model is to allow parameters c or b or both to be negative, abandoning the original framing of "costs" and "benefits" of agents using trait A. Doing so allows us to reach the other kinds of interactions described in Section 2 of the main text and general expression in main text Eq. (3), where $m = b + d$, $n = b$, and $k = c/d$.

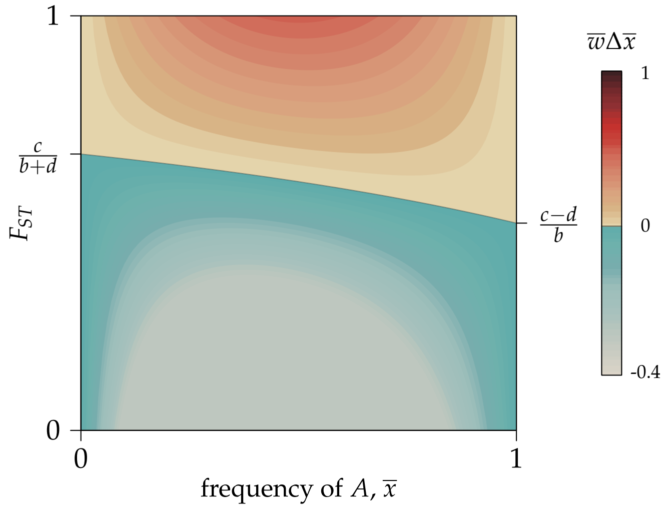

Figure A3: Contour levels (coloration) showing the strength of selection on spread of the cooperative trait A in a metapopulation playing Prisoner's-Dilemma-like cooperative dilemmas with positive or negative synergy. Altruism will spread at any frequency, $\overline{x}$, provided the metapopulation $F_{ST} > (c - d\overline{x})/(b + d(1 - \overline{x}))$.

2.2 Linear synergy in generic dyadic interactions

Main text Eq. (3) presents a general payoff expression for frequency-dependent interactions within a group-structured population. One way to motivate this expression is by adding synergy to a Prisoner's Dilemma, as described in the previous section. Another is to use generic terms for each payoff, a common convention is the letters R, S, T, P for the "Reward" of mutual cooperation, the "Sucker" payoff of cooperating against a defector, the "Temptation" to defect, and the "Punishment" of mutual defection (Allen & Nowak, 2015).

If we wish to avoid the narrow framing of "cooperation" and "defection", we may instead say that if two partners both choose strategy A, they both receive payoff a. If the focal player chooses A and the other chooses B, focal receives b and the other player receives c, and *vis versa* if focal chooses B and the other player A. If both players choose B, they both receive outcome d (note that the symbols b, c and d are used differently here compared to the PD). We can represent this with a 2x2 payoff matrix as payoffs to the Row player in Table A3.

	A	B
A	a	b
B	c	d

Table A3: Payoff matrix in a generic 2-player dyadic game.

For a population whose average tendency to play A is p, if any individual i plays strategy A x_i of the time, their expected payoff w_i would be

$$w_i = w_0 + x_i(pa + (1-p)b) + (1-x_i)(pc + (1-p)d) \tag{A8}$$

where w_0 gives a baseline payoff for all players. If we posit the existence of a population frequency $p = k$ at which all players have the same payoff, it must satisfy the equation

$$ka + (1-k)b = kc + (1-k)d$$

Solving for k gives

$$k = \frac{d-b}{(a-b)-(c-d)}$$

All frequency-dependent games have a k, except for the degenerate case where $(a-b) = (c-d)$. In this case, the system reduces to an additive game, and if $a < c$, gives the classic formulation of the dyadic one-shot Prisoner's Dilemma (SI Section 1.2).

Depending on the payoff structure, k may not lie between 0 and 1, but if it does, this represents a mixed equilibrium for the system, at which the balance of phenotypes causes all individuals to receive the same average payoff. Assuming such an equilibrium exists, we can fully express this system in terms of just three parameters. Define parameter m as the marginal effect of a within-group increase in trait A on the fitness of a focal individual with A, and n as the marginal effect of such an increase for a focal individual with trait B. For individuals with mixed strategies the marginal effect of an increase in A is $x_j m + (1-x_j)n$, the weighted average of m and n, and $\tilde{w}$ as the fitness payoff experienced by all players when $p = k$, which is both $w_0 + b + km$ and $w_0 + d + kn$. In these terms, fitness for individual i is

$$w_i = \tilde{w} + x_i m(p-k) + (1-x_i)n(p-k)$$

Assuming this fitness dynamic takes place within each group j of a metapopulation leads to main text Eq. (3).

2.3 Linear synergy in N-player interactions

We can also derive main text Eq. (3) from a model of N-person group interactions, in which each individual affects the payoffs of all others in a group simultaneously. Starting with a modified version of the PGG (SI Section 1.2), we begin by assuming that behavior A creates a "public good" via aggregate production function GX, where again $X = \sum_{i=1}^{N} x_i$, and each individual i experiences a "cost" $-Cx_i$ in proportion to their propensity towards A. To abstract away from the original cooperative framing of the PGG, we need not assume that multiplier G is positive, nor that $-C$ is negative; $G < 0$ implies that, as A increases in frequency within the group, some "public bad" is also increasing.

To create synergistic dynamics, we make two additional assumptions in the N-person context. First, both trait A and B generate exclusive "club good" payoffs available to players only as a function of their relative participation in each behavior (McKean, 2000; Peña, Nöldeke, & Lehmann, 2015). In a cultural context, this reflects how different norms can have frequency-dependent effects for their users (Boyd & Richerson, 2002). Mathematically, this assumption takes the form of two additional production functions, $S_A(X)$ and $S_B(X)$. Because these are club payoffs, individual i with phenotype x_i receives a share of $S_A(X)$ in proportion to their investment, x_i/X, so as a result their total fitness payoff is

$$w_i = w_0 + \frac{GX}{N} - Cx_i + \frac{S_A(X)x_i}{X} + \frac{S_B(X)(1 - x_i)}{N - X}$$

where again $p = X/N$, the mean frequency of trait A in the group. We can incorporate assumptions about the synergistic nature of social interactions using different mathematical forms of the production function (Ostrom, 2003). Assuming that the relative benefits for using a behavior are directly proportional to the number of other users of that behavior, the club production functions take the form $S_A(X) = PX^2$ and $S_B(X) = Q(1 - X)^2$ (Fig. A4, left). As with G, we make no assumptions about the signs or relative magnitudes of parameters P and Q; a positive production parameter indicates that each additional unit of investment into the behavior creates a larger and larger aggregate payoff, while a negative parameter indicates that each additional unit of investment is increasingly detrimental to participants, as in a market-entry game (Anderson & Engers, 2007). In both cases, the marginal effects are linear functions of X.

We can show that linear returns to scale on the aggregate level leads to constant per-capita returns to scale ($\lambda = 1$ in the geometric production functions of Peña et al. (2015)). The individual fitness expression in this model is

$$\begin{aligned} w_i &= w_0 + Gp - Cx_i + PXx_i + Q(1 - X)(1 - x_i) \\ &= w_0 + Gp - Cx_i + \frac{P}{N}px_i + \frac{Q}{N}(1 - p)(1 - x_i) \end{aligned}$$

Notably, even in the absence of any public good ($G = 0$), cost C still has meaning as the "entrance fee" to an individual for fully switching from one strategy to the other. Define trait frequency k at which all individuals in the group experience the same fitness $\tilde{w}$, which

may be achievable ($0 < k < 1$) or not ($k > 1$ or $k < 0$). At k, the fitness for a full A-type ($x_i = 1$) must equal that of a full B-type ($x_i = 0$), so

$$w_0 + Gk + \frac{Q}{N}(1-k) = w_0 + Gk - C + \frac{P}{N}k$$

$$k = \frac{Q + NC}{Q + P}$$

Note that $0 < k < 1$ implies that $C < P/N$ and $C < Q/N$ if $Q + P > 0$, or $C > P/N$ and $C > Q/N$ if $Q + P < 0$. The fitness payoff at this frequency is thus

$$\tilde{w} = w_0 + k\left(G - \frac{Q}{N}\right) + \frac{Q}{N}.$$

Rearranging individual fitness yields

$$w_i = \tilde{w} + (p-k)\left(G - \frac{Q}{N} + x_i\left(\frac{P}{N} + \frac{Q}{N}\right)\right)$$

$$= \tilde{w} + (p-k)\left(x_i\left(G + \frac{P}{N}\right) + (1-x_i)\left(G - \frac{Q}{N}\right)\right)$$

$$= \tilde{w} + (p-k)(x_i m + (1-x_i)n)$$

where $m = G + P/N$ and $n = G - Q/N$, both constants with respect to p, so we have again reached main text Eq. (3)(Fig. A4, right). We can also represent this model in the common b, c and d notation per Queller (1985), where $b = G - Q/N$, $c = C + Q/N$ and $d = (P + Q)/N$. This illustrates how both club production functions contribute to the synergistic term d.

The above model demonstrates that, although the assumption that interactions take place in dyads is sufficient (Van Cleve & Lehmann, 2013) and often quite intuitive, club goods with quadratic production functions can also lead to constant returns to scale (marginal effects) for individuals (Boyd & Richerson, 2002; Ostrom, 2003).

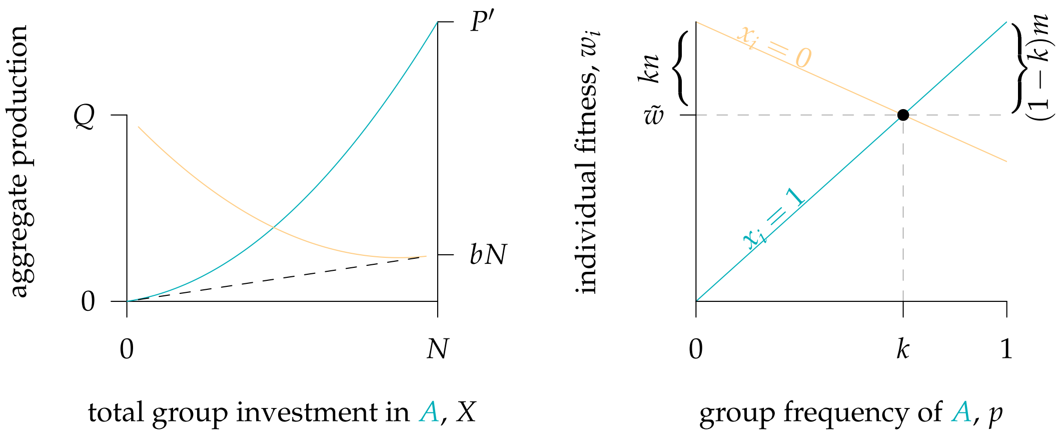

Figure A4: (left) Example aggregate production functions from the joint action of N individuals choosing strategy A at amount $X = \sum_{i=1}^{N} x_i$, from functions $S_B(X)$ (yellow) and $S_A(X) + GX$ (green) exhibiting linear returns to scale. Payoff $P' = P + GN$ represents the total output with all individuals using behavior A, and Q the total output with all individuals using behavior B. The example here shows $P = 500$, $Q = 400$, $G = 1$, and $C = 2$. (right) Individual fitness payoffs corresponding to the aggregate payoff functions shown, all terms m, n, k and $\tilde{w}$ calculated according to the N-person model in SI Section 2.2. Note that $0 < k < 1$ implies that $C < P/N$ and $C < Q/N$ if $Q + P > 0$, or $C > P/N$ and $C > Q/N$ if $Q + P < 0$.

3 Four Essential Frequency-dependent Games

Here, we review four synergistic games discussed in the main text.

3.1 Stag Hunt

The Stag Hunt is a classic model of social coordination (Skyrms, 2004) in which two hunters can together catch a stag, or individually secure a hare. Both hunters are better off if they can coordinate on the Stag strategy, but each runs the risk of ending up with nothing if they try for the stag while their partner chooses a hare. Formally, we can say a Hare player receives payoff H regardless of their partner's choice, while a Stag player either receives $S > H > 0$ if their partner also plays Stag, or 0 if their partner plays Hare. Thus, we can define a Stag Hunt using the symmetric row-player payoff matrix (Table A4).

In an evolutionary context, we say any specific individual i has a behavioral phenotype that plays Stag x_i of the time, and in aggregate the population frequency of Stag play is p. Assuming random pairing, the expected fitness payoff to individuals is then

$$w_i = w_0 + x_i p S + (1 - x_i) H \tag{A9}$$

	Stag	Hare
Stag	S	0
Hare	H	H

Table A4: Payoff matrix in a 2-player dyadic Stag Hunt.

for some baseline fitness w_0 shared by all members of the population. At Stag frequency $k = H/S$, all individuals have the same average fitness $\tilde{w} = w_0 + H$, so this is the mixed equilibrium of the evolutionary system. If $p > k$, the fitness payoff for Stag players is higher than the payoff for Hare players, while if $p < k$, the reverse is true (Fig. A5, left). The mixed equilibrium is thus unstable, and selection generally leads the population to coordinate entirely on one ($p = 1$), or the other ($p = 0$) strategy.

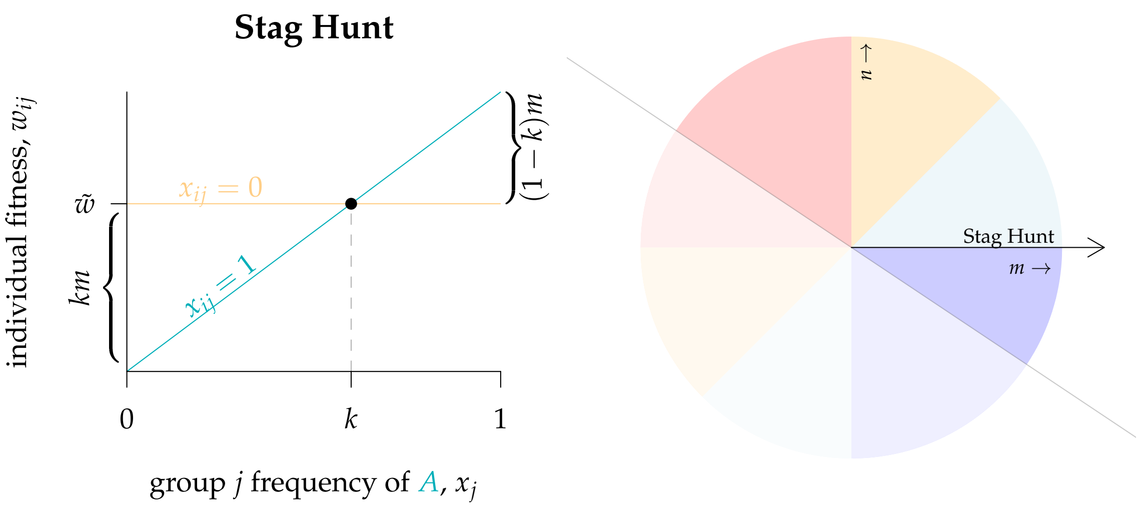

Figure A5: (left) Fitness functions for Stag-Hunt-like interactions per main text Eq. (3), where $k = 0.6$. (right) Line segment in (m, n) space corresponding to Stag Hunt interactions ($\theta = 0$ if trait A is Stag).

The coordination dynamic of the Stag Hunt can be explored in the context of our generic metapopulation model of linear synergy (Eq. (3)) by interpreting strategy A as "Stag" and strategy B as "Hare", so that $m = S$ and $n = 0$. In (m, n) space, Stag-Hunt-like interactions lie along the line segment given by $n = 0$, where $m > 0$. In radians, these interactions are therefore defined along angle $\theta = 0$ (Fig. A5, right).

Applying these constraints to main text Eq. (5), the expected change in the prevalence of A in the metapopulation simplifies to

$$\overline{w}\Delta\overline{x} = m\overline{x}(1-\overline{x})\big((\overline{x}-k) - F_{ST}(\overline{x}-1)\big). \tag{A10}$$

This equation can be described by a contour diagram over possible values of $\overline{x}$ and F_{ST}

(Fig. A6). Selection moves the metapopulation towards the A-equilibrium whenever the metapopulation mean frequency $\overline{x} > k$ regardless of the F_{ST} variance ratio. This follows the basic coordination pattern, which favors common strategies. When $\overline{x} < k$, selection will still favor the Stag strategy provided that it is concentrated within groups above threshold $F_{ST} = (k - \overline{x})/(1 - \overline{x})$. Below this threshold, Stag strategies are too widely dispersed to effectively coordinate, and the metapopulation moves towards a state of all-Hare. In the extreme scenario where $\overline{x} \approx 0$, Stag can still invade a metapopulation of Hare provided that $F_{ST} > k$.

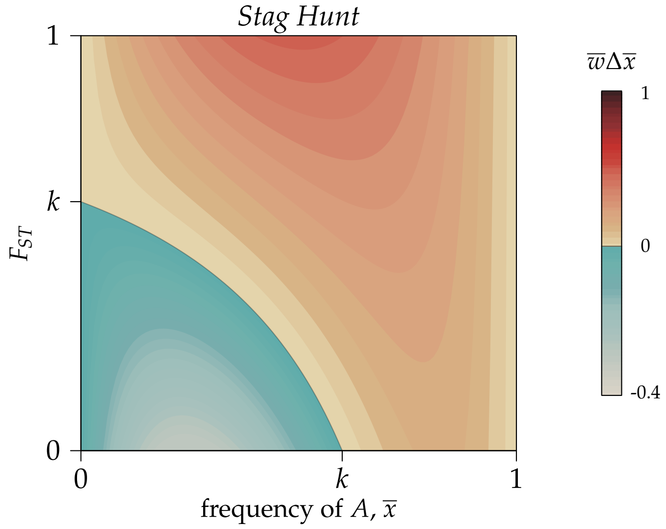

Figure A6: Contour levels (coloration) showing the strength of selection on spread of the group-beneficial trait A in a metapopulation playing Stag-Hunt-like coordination dilemmas per Equation (A10). Stag behavior can increase at any frequency, $\overline{x}$, provided that metapopulation $F_{ST} > (k - \overline{x})/(1 - \overline{x})$.

Taking the derivative of Equation (A10) with respect to F_{ST} yields

$$\mathrm{d}\overline{w}\Delta\overline{x}/\mathrm{d}F_{ST} = m\overline{x}(1 - \overline{x})^2 \tag{A11}$$

which is necessarily positive; a marginal increase in F_{ST} within a Stag-Hunt-like interaction always favors the spread of group-beneficial behaviors. In this regard, it is like an additive model of altruism such as the Prisoner's Dilemma. However, it differs from the Prisoner's Dilemma because if Stag is already common enough within a metapopulation (greater than k), it can stabilize at *any* level of within-group diversity.

The Stag Hunt is also notable because, although $x_j = 0$ has the lower pure-strategy group payoff, group fitness is minimized at frequency $x_j^* = k/2$, half-way to the mixed equilibrium. Thus, a group moving out of the Hare basin of attraction must undergo a decrease in average fitness before realizing the larger benefits from the presence of Stag behavior. This limits the force of equilibrium selection between groups of Stag and groups of Hare, compared to coordination games in which $n > 0$ and group-beneficial strategies produce an additional net benefit to all members of the group (Boyd & Richerson, 2002).

3.2 Hawk-Dove

The Hawk-Dove game was initially described by Maynard Smith and Price (1973) and has become a standard model of conflict and competition. In this game, we imagine a pair of agents disputing access to some resource. If both employ the Dove strategy, each has an equal chance of getting the resource. If one plays Hawk and the other Dove, the Hawk gets all of the resource without a fight, and the Dove nothing. If both play Hawk, though, a fight begins in which one gains the resource at a large cost to the other, again with equal chance to each participant. Let V be the total value of the resource under dispute, and $C > V$ represent the cost of losing a fight, if both players choose Hawk. This game structure then implies the symmetric row-player payoff matrix in Table A5.

	Dove	Hawk
Dove	$V/2$	0
Hawk	V	$(V-C)/2$

Table A5: Payoff matrix in a 2-player dyadic Stag-Hunt.

In an evolutionary context, we say any specific individual i with a behavioral phenotype that plays Dove x_i of the time, and in aggregate the population frequency of Dove play is p. Assuming random pairing, the expected fitness payoff to each individual is then

$$w_i = w_0 + x_i pV/2 + (1 - x_i)(pV + (1 - p)(V - C)/2) \tag{A12}$$

where baseline fitness w_0 is shared by all members of the population. At Dove frequency $k = (C - V)/C$, all individuals have the same average fitness $\bar{w} = w_0 + kV/2$, so this is the mixed equilibrium of the evolutionary system, and in an anti-coordination game dynamic, this mixed equilibrium is also the only stable one, as each strategy enjoys a higher payoff when rare and so can invade the other.

The anti-coordination dynamic of the Hawk-Dove can be explored in the context of our generic metapopulation model of linear synergy (Eq. (3)) by interpreting strategy A as "Dove" and strategy B as "Hawk", so that $m = V/2$ and $n = (V + C)/2$ (Fig. A7). Rearranging the definition of k in the Hawk-Dove game gives

$$k = \frac{n - 2m}{n - m}$$

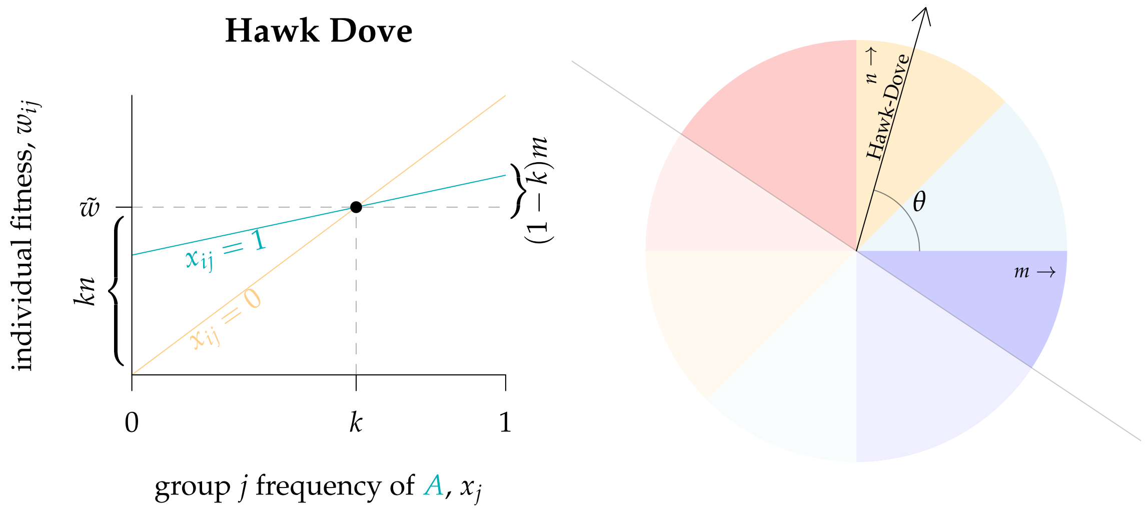

Figure A7: (left) Fitness functions for Hawk-Dove-like interactions per main text Eq. (3), where $k = 0.6$. (right) Line segment in (m, n) space corresponding to Hawk-Dove interactions ($\theta = 1.29$ if trait A is Dove).

meaning that, in (m, n) space, Hawk-Dove-like interactions lie along the line segment given by

$$n = m\left(\frac{2-k}{1-k}\right)$$

where $n > 0$ and $m > 0$. In radians, these interactions are defined along angle $\theta = \text{atan2}(2-k, 1-k)$. Applying these constraints to main text Eq. (5), the expected change in the prevalence of A in the metapopulation simplifies to

$$\overline{w}\Delta\overline{x} = (m-n)\overline{x}(1-\overline{x})\big((\overline{x}-k) - F_{ST}(\overline{x}-k+1)\big). \tag{A13}$$

This equation can be described by a contour diagram over possible values of $\overline{x}$ and F_{ST} (Fig. A8). Selection increases the frequency of Dove whenever the metapopulation mean frequency $\overline{x} < k$ regardless of the F_{ST} variance ratio. This follows the basic anti-coordination pattern, which favors rare strategies. When $\overline{x} > k$, selection will still favor the Dove strategy provided that it is concentrated within groups above threshold $F_{ST} = (\overline{x}-k)/(\overline{x}-k+1)$. Below this threshold, Dove strategies are too widely dispersed to effectively prevent Hawks from invading, and the metapopulation moves towards the mixed equilibrium. In the extreme scenario where $\overline{x} \approx 1$, Hawks can yet be prevented from invading provided that $F_{ST} > (1-k)/(2-k)$, e.g. if Hawks can be effectively quarantined within a single group.

Taking the derivative of Equation (A13) with respect to F_{ST} yields

$$\mathrm{d}\overline{w}\Delta\overline{x}/\mathrm{d}F_{ST} = -(m-n)\overline{x}(1-\overline{x})\big((\overline{x}-k)+1\big) \tag{A14}$$

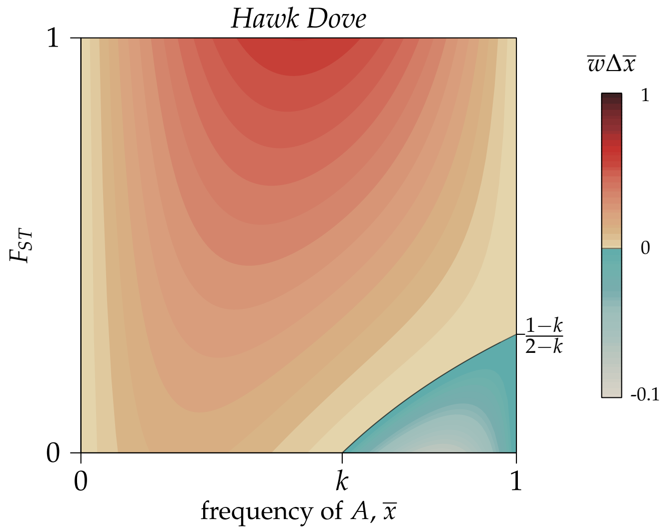

Figure A8: Contour levels (coloration) showing the strength of selection on spread of the group-beneficial trait A in a metapopulation playing Hawk-Dove-like anti-coordination dilemmas per Equation (A12). Dove behavior can increase at any frequency, $\overline{x}$, provided that metapopulation $F_{ST} > (1-k)/(2-k)$.

which is necessarily positive; a marginal increase in F_{ST} within a Hawk-Dove-like interaction always favors the spread of group-beneficial behaviors. In this regard, it is like an additive model of altruism such as the Prisoner's Dilemma. However, it differs from the Prisoner's Dilemma because group-beneficial norms can stabilize at *any* level of within-group diversity under certain conditions.

In a Hawk-Dove game, the group average fitness is given by

$$w_j = \tilde{w} + (2-k)(x_j - k) - (x_j - k)x_j \tag{A15}$$

which is maximized when $x_j = 1$. A marginal increase in Hawks always lowers group fitness. This is also true more generally in anti-coordination dilemmas in which $m > 0$ and $m < n < m(2-k)/(1-k)$, but *not* true if $m > 0$ and $n > m(2-k)/(1-k)$. As such, the Hawk-Dove is an important transition point between interactions in which the presence of B is uniformly deleterious and those in which some small amount of B can increase group fitness.

3.3 Invisible Hand

The Invisible Hand game takes its name from Adam Smith's famous metaphor for the unintended group benefits of specialization and exchange. Consider, for example, a game in which a pair in a foraging party either can construct shelter or search for food. The payoff table here is given by Table A6.

	Shelter	Food
Shelter	H	$H + E/2$
Food	$H + E/2$	E

Table A6: Payoff matrix in a 2-player dyadic Invisible Hand.

If both players obtain food, they have no shelter. If both players each make a shelter, they have no food. As an anti-coordination game, individuals benefit from not doing the same thing.

In an evolutionary context, we can say any specific individual i has a behavioral phenotype that plays Food x_i of the time, and in aggregate the population frequency of Food play is p. Assuming random pairing, the expected fitness payoff to individuals is then

$$w_i = w_0 + x_i(pE + (1-p)(E/2 + H)) + (1 - x_i)(p(E/2 + H) + (1-p)H) \quad \text{(A16)}$$

for some baseline fitness w_0 shared by all members of the population. At Food frequency $k = E/2H$, all individuals have the same average fitness $\bar{w} = w_0 + kE/2 + H$, so this is the mixed equilibrium of the evolutionary system, and in an anti-coordination game dynamic, this mixed equilibrium is also the only stable one, as each strategy enjoys a higher payoff when rare and so can invade the other. Unlike anti-coordination dilemmas like Hawk-Dove, though, both are better off as a result.

The anti-coordination dynamic of the Invisible Hand game can be explored in the context of our generic metapopulation model of linear synergy (Eq. (3)) by interpreting strategy A as "Food" and strategy B as "Shelter", so $n = E/2$ and $m = E/2 - H$ (Fig. A9). Rearranging the definition of k in the Invisible Hand game gives

$$k = \frac{n}{n-m}$$

meaning that, in (m, n) space, Invisible-Hand-like interactions lie along the line segment given by

$$n = -m\left(\frac{k}{1-k}\right)$$

where $n > 0$ and $m < 0$. In radians, these interactions are defined along angle $\theta = \text{atan2}(k, -(1-k))$. Applying these constraints to the main text Eq. (5), the expected change in the prevalence of A in the metapopulation simplifies to

$$\overline{w}\Delta\overline{x} = (m-n)\overline{x}(1-\overline{x})\big((\overline{x}-k) - F_{ST}(\overline{x}-(1-k))\big) \quad \text{(A17)}$$

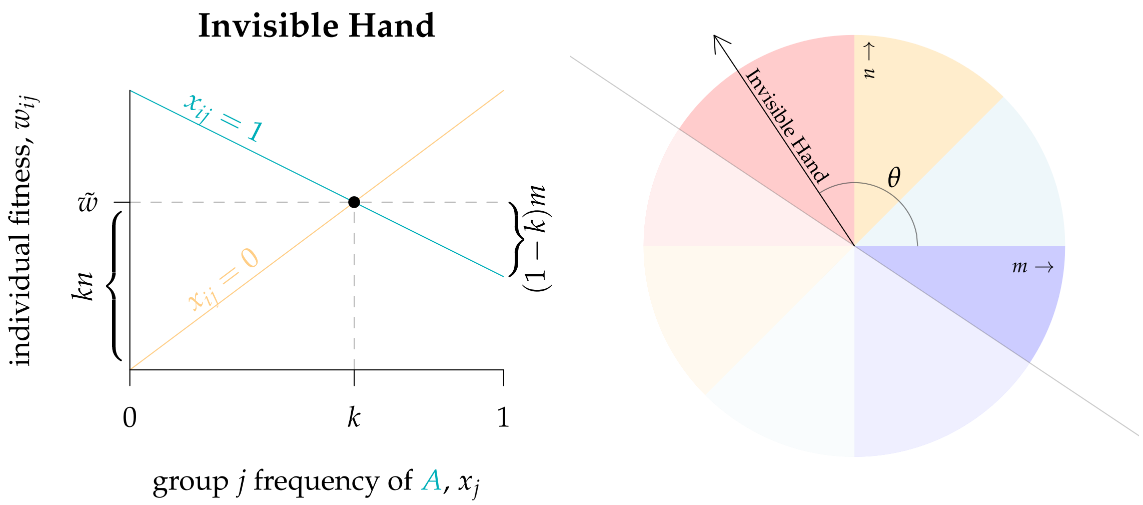

Figure A9: (left) Fitness functions for Invisible-Hand-like interactions per main text Eq. (3), where $k = 0.6$. Note that in Invisible Hand $k > 0.5$ is required for A to be GBT. (right) Line segment in (m, n) space corresponding to Invisible Hand interactions ($\theta = 2.16$).

The dynamics of this equation can be described by a contour diagram over possible values of $\overline{x}$ and F_{ST} (Fig. A10). As in the Hawk-Dove and other anti-coordination games, selection favors group-beneficial behaviors whenever the metapopulation mean frequency $\overline{x} < k$, at any variance ratio of F_{ST}. When $\overline{x} > k$, selection will still favor strategy A provided that it is concentrated within groups above threshold $F_{ST} = (\overline{x} - k)/(\overline{x} - (1-k)$. In the extreme scenario where $\overline{x} \approx 1$, B can be prevented from spreading provided that $F_{ST} > (1-k)/k$.

Taking the derivative of Eq. A17 gives

$$\mathrm{d}\overline{w}\Delta\overline{x}/\mathrm{d}F_{ST} = -(m-n)\overline{x}(1-\overline{x})(\overline{x}-(1-k)) \qquad \text{(A18)}$$

which equals 0 if $\overline{x} = 1-k$. If $\overline{x} < (1-k)$, the marginal effect of an increase in F_{ST} on selection for the A is negative, while if $\overline{x} > (1-k)$, this effect is positive. Even when the marginal effect of F_{ST} is positive, this does not necessarily enhance group fitness; on a group level, the average payoff is

$$w_j = \tilde{w} + (m-n)(x_j - k)^2$$

which implies a group-fitness maximum at $x_j^* = k$. Because group fitness is maximized at the mixed equilibrium in the Invisible Hand game, the effect of F_{ST} here differs from Stag Hunt and Hawk-Dove. When $\overline{x} < (1-k)$, A-players are welcome additions to groups mostly composed of trait B, and A will spread at any frequency. However, the marginal effect of F_{ST} in this region is negative. When $\overline{x} > k$, the marginal effect of F_{ST} on $\Delta\overline{x}$ is positive, but this has the effect of preserving A in highly-segregated groups and preventing the appearance of mixed-strategy groups which realize the highest payoffs. In both cases,

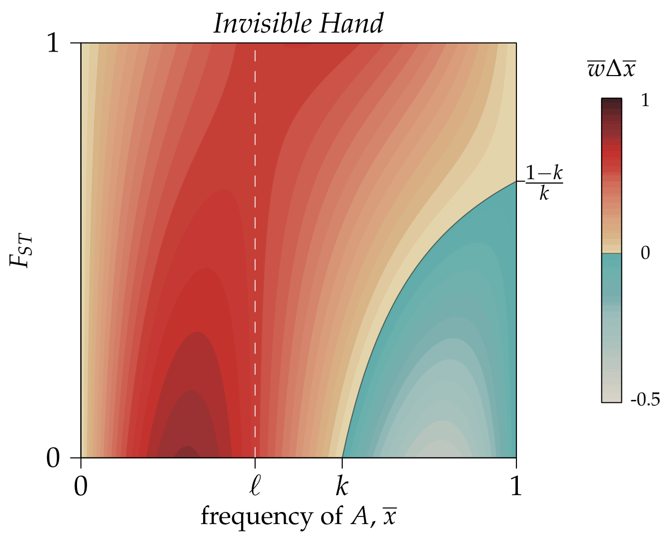

Figure A10: Contour levels (coloration) showing the strength of selection on spread of the group-beneficial trait A in a metapopulation playing Invisible-Hand-like anti-coordination dilemmas per Equation (A17). Strategy A can increase at any frequency, $\overline{x}$, provided that metapopulation $F_{ST} > (\overline{x} - k)/(\overline{x} - \ell)$. Invisible Hand has a negative marginal effect of F_{ST} below, and positive marginal effect above, the frequency $\overline{x} = 1 - k$ (white dashed line).

greater segregation between strategies slows the spread of group-beneficial A's through the metapopulation; F_{ST} functions to hinder, rather than assist, movement towards a social optimum within the population. This phenomenon is present in complementary games more generally, and also in some anti-coordination dilemmas.

3.4 Pure Coordination

In a Pure Coordination game, players must choose between two equivalent behavioral options that yield the same payoff regardless of which they coordinate on. If they fail to coordinate, and each chooses a different behavior, they both experience a cost (which may or may not be the same for each player). The Pure Coordination game is 'pure' because each alternative is functionally equivalent if both players use it. Perhaps the most common real-world example of a coordination dilemma is bidirectional traffic flows. Travellers on roads, bike paths, walkways, etc. can either stay to the right or left of oncoming traffic, and are generally much better off so long as all travellers agree on which side is correct.

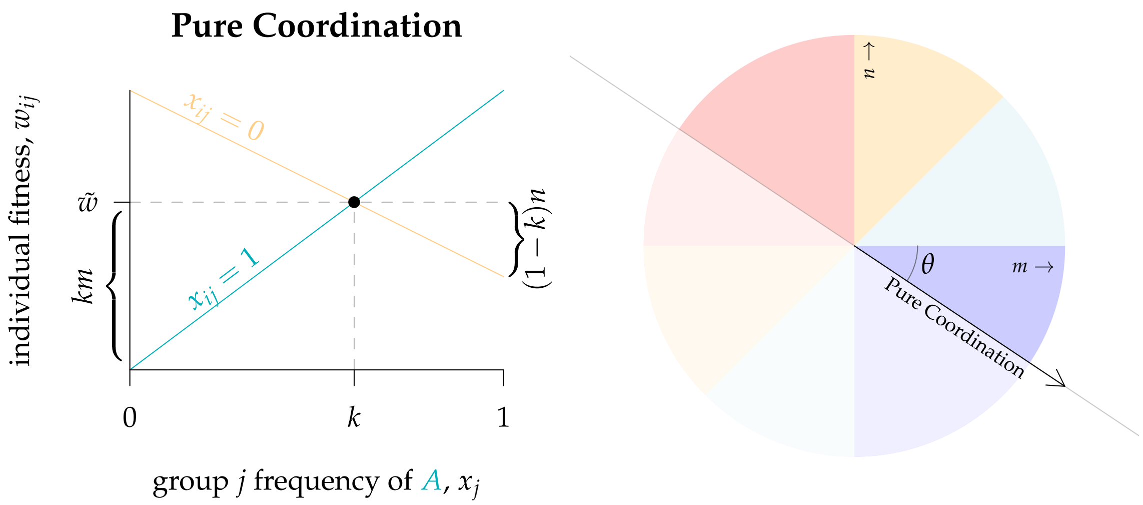

Figure A11: (left) Fitness functions for Pure-Coordination-like interactions per main text Eq. (3), where $k = 0.6$. Note that neither trait is GBT in this game. (right) Line segment in (m, n) space corresponding to Pure Coordination interactions ($\theta = -0.58$).

Imagine two travellers must each decide to either move Left or Right when passing each other on a trail. Define payoff b as the outcome each player receives for successful coordination. If the row player chooses Left while the column player chooses Right, row receives $-C$ and column receives $-D$, and vis versa if the row player chooses Right and the column player Left. Without loss of generality, we can simplify the system by setting $b = 0$. The resulting payoff matrix is given by Table A7.

	Left	Right
Left	0	$-C$
Right	$-D$	0

Table A7: Payoff matrix in a 2-player dyadic Pure Coordination game.

For our purposes, the definition of the Pure Coordination game requires only that Left-Left and Right-Right pairs receive the *same* payoff, so that no GBT exists. It does not matter if $C > D$, $C < D$, or $C = D$.

In an evolutionary context, we can say that any specific individual i has a behavioral phenotype that plays Left x_i of the time, and in aggregate the population frequency of Left play is p. Assuming random pairing, the expected fitness payoff is

$$w_i = w_0 - x_i(1-p)C - (1-x_i)pD \tag{A19}$$

for some baseline fitness w_0 shared by all members of the population. At Left frequency $k = C/(D + C)$ all individuals have the same average fitness $\tilde{w} = w_0 - Dk$, so this is

the mixed equilibrium of the evolutionary system. As this is a coordination game, the equilibrium is unstable, and the system will quickly move to either all Right or all Left strategies.

The coordination dynamic of a Pure Coordination game can be explored in the context of our generic metapopulation model of linear synergy (Eq. (3)) by interpreting strategy A as "Left" and strategy B as "Right", so that $m = C$ and $n = -D$ (Fig. A11). Rearranging the definition of k in the Pure Coordination game gives

$$\frac{n}{m} = -\frac{1-k}{k}$$

meaning that, in (m, n) space, Pure-Coordination-like interactions lie along the line segment given by

$$n = -m\left(\frac{1-k}{k}\right)$$

where $m > 0$ and $n < 0$. In radians, these interactions are defined along angle $\theta = \text{atan2}(-(1-k), k)$. Applying these constraints to main text Eq. (5), the expected change in the prevalence of A in the metapopulation simplifies to

$$\overline{w}\Delta\overline{x} = (m-n)\overline{x}(1-\overline{x})(\overline{x}-k)(1-F_{ST}) \tag{A20}$$

This equation can be described by a contour diagram over possible values of $\overline{x}$ and F_{ST} (Fig. A12). Selection favors behavior A whenever the metapopulation mean frequency $\overline{x} > k$, and B when $\overline{x} < k$, at any variance ratio of F_{ST}. In any metapopulation for which $\overline{x} = k$, the system is in an unstable equilibrium.

Taking the derivative of (A20) with respect to F_{ST} yields

$$\mathrm{d}\overline{w}\Delta\overline{x}/\mathrm{d}F_{ST} = -(m-n)\overline{x}(1-\overline{x})(\overline{x}-k) \tag{A21}$$

which is necessarily negative. Thus, unlike in the evolution of altruism, or in the Hawk-Dove or Stag Hunt, a marginal increase in F_{ST} within a Pure-Coordination-like interaction always slows the rate at which the population reaches a payoff-optimal pure strategy equilibrium.

The Pure Coordination interaction represents an extreme boundary of interactions considered here, because each pure-strategy equilibrium has the same average fitness. Here, then, the definition of A and B in our frequency-dependent model is truly arbitrary and neither is the GBT. The non-coordination costs determine the location of the unstable mixed equilibrium, but in Pure Coordination, the worst outcome for a group is always a 50/50 split between the two behaviors.

Simple coordination games that are *near* Pure Coordination show more complex evolutionary dynamics. These games, which we might call "Impure Coordination", exist when $m > 0$ and $0 > n > -m(1-k)/k$. A representative example is shown in Fig. A13, which mirrors the structure of Invisible Hand. This game was initially described by (Allen & Nowak, 2015).

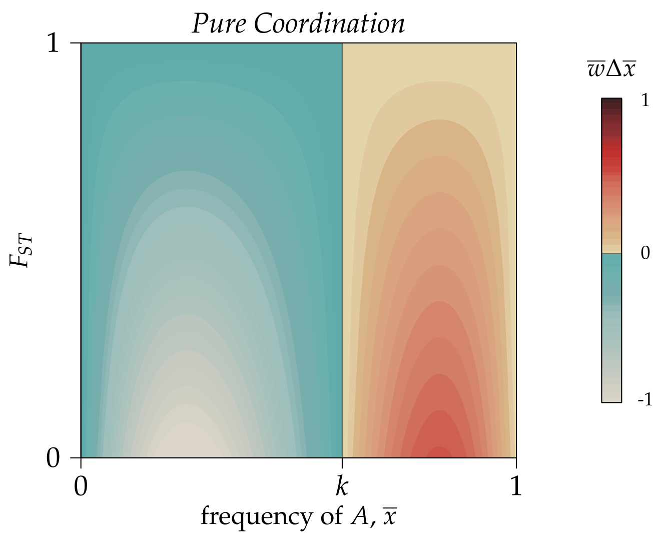

Figure A12: Contour levels (coloration) showing the strength of selection on spread of the group-beneficial trait A in a metapopulation playing Pure Coordination-like coordination dilemmas per (A20). Strategy A can increase at any F_{ST} provided that $\overline{x} > k$.

References

Allen, B., & Nowak, M. A. (2015). Games among relatives revisited. *Journal of Theoretical Biology*, *378*, 103–116. doi: 10.1016/j.jtbi.2015.04.031

Anderson, S. P., & Engers, M. (2007). Participation games: Market entry, coordination, and the beautiful blonde. *Journal of Economic Behavior & Organization*, *63*(1), 120–137. doi: 10.1016/j.jebo.2005.05.006

Boyd, R., & Richerson, P. J. (2002). Group Beneficial Norms Can Spread Rapidly in a Structured Population. *Journal of Theoretical Biology*, *215*(3), 287–296. doi: 10.1006/jtbi.2001.2515

Gavrilets, S., & Richerson, P. J. (2017). Collective action and the evolution of social norm internalization. *Proceedings of the National Academy of Sciences*, *114*(23), 6068–6073. doi: 10.1073/pnas.1703857114

Hamilton, W. D. (1975). Innate social aptitudes of man: an approach from evolutionary genetics. In R. Fox (Ed.), *Biosocial Anthropology* (pp. 133–155). Malaby Press, London.

Maynard Smith, J., & Price, G. R. (1973). The logic of animal conflict. *Nature*, *146*, 15–18.

McKean, M. A. (2000). Common Property: What Is It, What Is It Good for, and What Makes

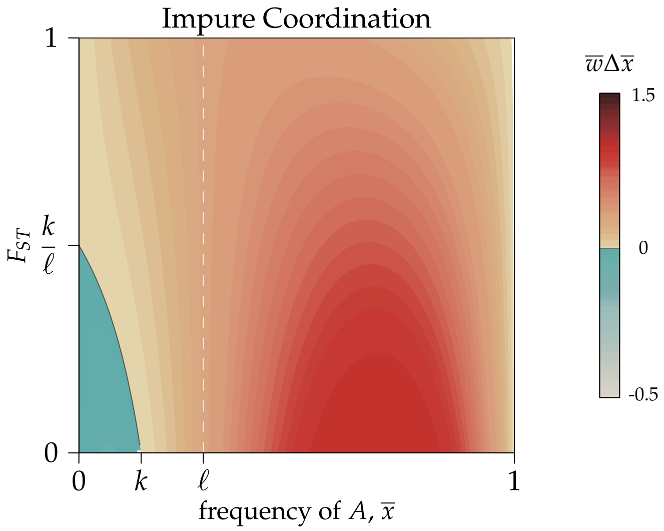

Figure A13: Contour levels (coloration) showing the strength of selection on spread of the group-beneficial trait A in a metapopulation playing the simple coordination game described by Allen and Nowak (2015) (see their Fig. 3 and Eq. (12)) in which $k = 1/7, m = 2, n = -5$. Strategy A can increase at any frequency, $\overline{x}$, provided that metapopulation F_{ST} exceeds the critical value set by main text Eq. (6). The gradient describing the effect of F_{ST} on selection reverses direction at frequency $\overline{x} = \ell = m/(m-n)$ (white dashed line) per Eq. (8).

It Work? In C. C. Gibson, M. A. McKean, & E. Ostrom (Eds.), *People and Forests* (pp. 27–56). The MIT Press. doi: 10.7551/mitpress/5286.003.0008

Ostrom, E. (2003). How Types of Goods and Property Rights Jointly Affect Collective Action. *Journal of Theoretical Politics*, *15*(3), 239–270. doi: 10.1177/0951692803015003002

Peña, J., Nöldeke, G., & Lehmann, L. (2015). Evolutionary dynamics of collective action in spatially structured populations. *Journal of Theoretical Biology*, *382*, 122–136. doi: 10.1016/j.jtbi.2015.06.039

Queller, D. C. (1985). Kinship, reciprocity and synergism in the evolution of social behaviour. *Nature*, *318*(6044), 366–367. doi: 10.1038/318366a0

Skyrms, B. (2004). *The Stag Hunt and the Evolution of Social Structure*. Cambridge: Cambridge University Press.

Taylor, C., & Nowak, M. A. (2007). Transforming the dilemma. *Evolution*, *61*(10), 2281–2292. doi: 10.1111/j.1558-5646.2007.00196.x

Van Cleve, J., & Lehmann, L. (2013). Stochastic stability and the evolution of coordination

in spatially structured populations. *Theoretical Population Biology*, *89*, 75–87. doi: 10.1016/j.tpb.2013.08.006
